# Halo-Substituted Chalcones and Azachalcones Inhibited Lipopolysaccharited-Stimulated Pro-Inflammatory Responses through the TLR4-Mediated Pathway

**DOI:** 10.3390/molecules23030597

**Published:** 2018-03-07

**Authors:** Tzenge-Lien Shih, Ming-Hwa Liu, Chia-Wai Li, Chia-Feng Kuo

**Affiliations:** 1 Department of Chemistry, Tamkang University, Tamsui Dist., New Taipei City 251, Taiwan; tlshih@mail.tku.edu.tw (T.-L.S.); 602180076@s02.tku.edu.tw (C.-W.L.); 2 Department of Food Science, Nutrition, and Nutraceutical Biotechnology, Shih Chien University, Zhongshan Dist., Taipei 104, Taiwan; a0141011@g2.usc.edu.tw

**Keywords:** azachalcones, chalcones, anti-inflammation, toll-like receptor 4

## Abstract

A series of B-ring, halo-substituted chalcones and azachalcones were synthesized to evaluate and compare their anti-inflammatory activity. Mouse BALB/c macrophage RAW 264.7 were pre-treated with 10 μg/mL of each compound for one hour before induction of inflammation by lipopolysaccharide (1 μg/mL) for 6 h. Some halo-chalcones and -azachalcones suppressed expression of pro-inflammatory factors toll-like receptor 4 (TLR4), IκB-α, transcription factor p65, interleukine 1β (IL-1β), IL-6, tumor necrosis factor α (TNF-α), and cyclooxygenase 2 (COX-2). The present results showed that the synthetic halo-azachalcones exhibited more significant inhibition than halo-chalcones. Therefore, the nitrogen atom in this series of azachalcones must play a more crucial role than the corresponding C-2 hydroxyl group of chalcones in biological activity. Our findings will lay the background for the future development of anti-inflammatory nutraceuticals.

## 1. Introduction

Inflammation is an immune response to injury and infection. Inflammation is considered to be beneficial when it is short term and under control; however, chronic production of radicals by inflammation causes oxidative damages and leads to chronic diseases [1,2,3,4,5]. Natural products that possess anti-inflammatory properties are valuable phytomedicine.

Chalcones belong to the flavonoid family. They are widely distributed in natural products and exhibit a variety of biological activities, including anti-oxidation, anti-inflammation, chemoprevention, cardioprotection, antidiabetics, and neuroprotection [6,7,8,9,10,11,12,13,14]. Novel chalcones with biological activities have been designed and synthesized [15,16,17,18,19,20,21].

**Figure 1:**
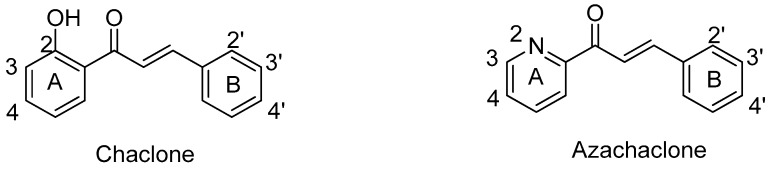
Structures of chalcones and azachalcones.

The skeleton of chalcones possesses a 1,3-diphenyl-2*E*-propene-1-one framework (Figure 1). The hydroxy and methoxy groups are the most common substituted groups on either or both rings in chalcones that show good antitubulin and cytotoxic activity [22]. Among them, the nearby C2′ hydroxyl moiety in ring A contributed hydrogen bonding to the carbonyl group, leading to the stabilization of the planar structure [23]. The most common strategy for the synthesis of chalcones is Claisen-Schmidt condensation [24,25].

Azachalcone is another class of the chalcone family in which the carbons of either rings A or B or both were replaced with a nitrogen atom. Because of their structural similarity, azachalcones were synthesized with the same strategy as for chalcones, in which the substituted acetophenone was replaced with 2-acetyl pyridine [26,27]. Studies have shown that azachalcones possess anti-bacterial, anti-inflammatory, and anti-cancer properties [28,29,30,31]. Azachalcones also served as precursors for synthesis of pyrazolines and pyrazoles [32].

**Figure 2:**
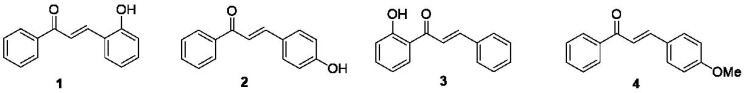
Natural chalcones **1**–**4**.

The halo-substituted chalcones and azachalcones are not naturally occurring compounds [33]. The halogen substitution in ring B of chalcones alters the electron distribution owing to its high electronegativity and dipole moment, which can influence the biological activity. The aim of this study is to synthesize novel, monohalogen-substituted chalcones and azachalcones in various positions in ring B and evaluate their anti-inflammatory activity. To the best of our knowledge, this article is the first report in a systematic study of the biological activities of monohalogen-substituted chalcones (**7a**–**i**) and azachalcones (**9a**–**i**) together (*vide infra*). The anti-inflammatory activities of synthetic chalcones and azachalcones were also compared with those of synthetic, naturally occurring chalcones **1**–**4** (Figure 2), which were synthesized; their spectroscopic data were all in agreement with the reported values.

## 2. Results and Discussion

### 2.1. Synthesis of Halo-Substituted Chalcones ***7a***–***i*** and Azachalcones ***9a***–***i***

**Figure 3:**
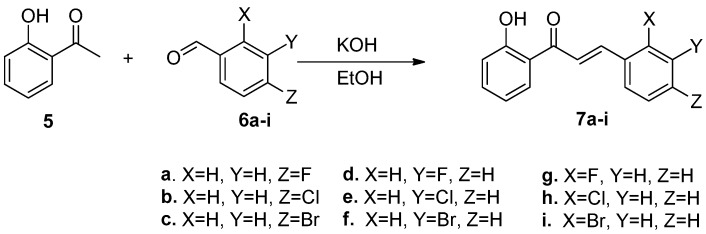
Claisen-Schmidt condensation in synthesis of chalcones **7a**–**i**.

In order to optimize the reaction condition, we repeated the synthesis of a series of halo-substituted chalcones **7g**–**i** from **5** and halobenzaldehyde **6a**–**i** by Claisen-Schmidt condensation (Figure 3). The optimized yields were listed in Table 1.

**Figure 4:**
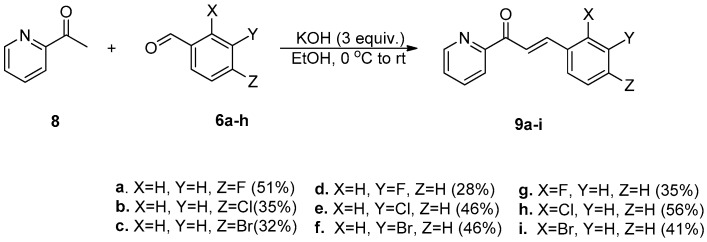
Conditions of synthesis of azachalcones **9a**–**i**.

**Figure 5:**
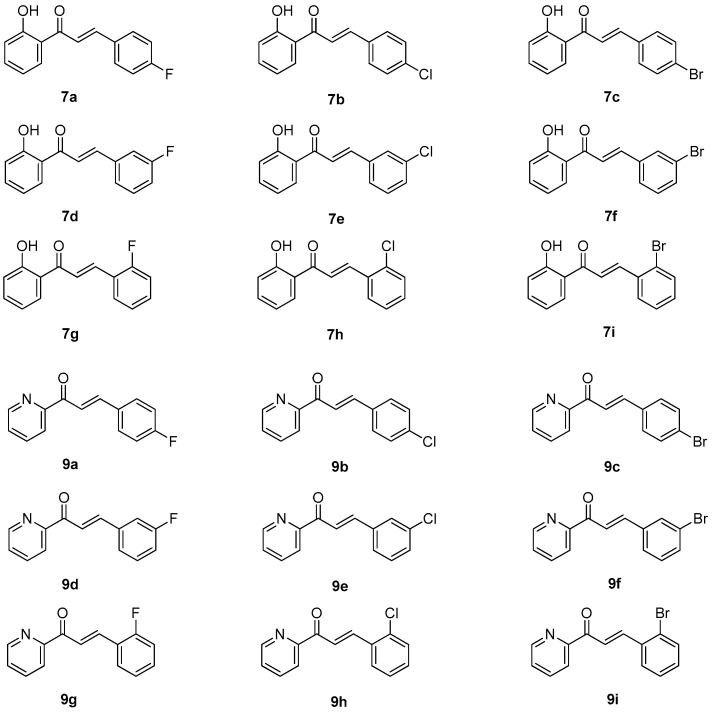
Chemical structures of synthetic chalcones **7a**–**i** and azachalcones **9a**–**i** in this study.

Azachalcones **9a**–**i** were synthesized from **8** and corresponding **6a**–**h** (Figure 4) as reported recently [33]. The structures of synthetic chalcones **7a**–**i** and azachalcones **9a**–**i** are listed in Figure 5. The ^1^H and ^13^C NMR data for compounds **7a**–**i** and **9a**–**i** are available as supporting materials.

### 2.2. Biological Evaluation

#### 2.2.1. Cytotoxic Effect of Halo-Substituted Chalcones (**7a**–**i**) and Azachalcones (**9a**–**i**)

To investigate the cytotoxicity of halo-substituted chalcones **7a**–**i**, azachalcones **9a**–**i**, and synthetic natural products **1**–**4**, the RAW 264.7 cells were treated with various concentrations of each compound for 24 h. The viable cell numbers were assayed and compared to the control group. According to the results in Table 2, 10 μg/mL for each compound was chose to treat RAW 264.7 for the evaluation of anti-inflammatory activities.

#### 2.2.2. Effect of Halo-Substituted Chalcones (**7a**–**i**) and Azachalcones (**9a**–**i**) on mRNA and Protein Expression of Pro-Inflammatory Factors

**Figure 6:**
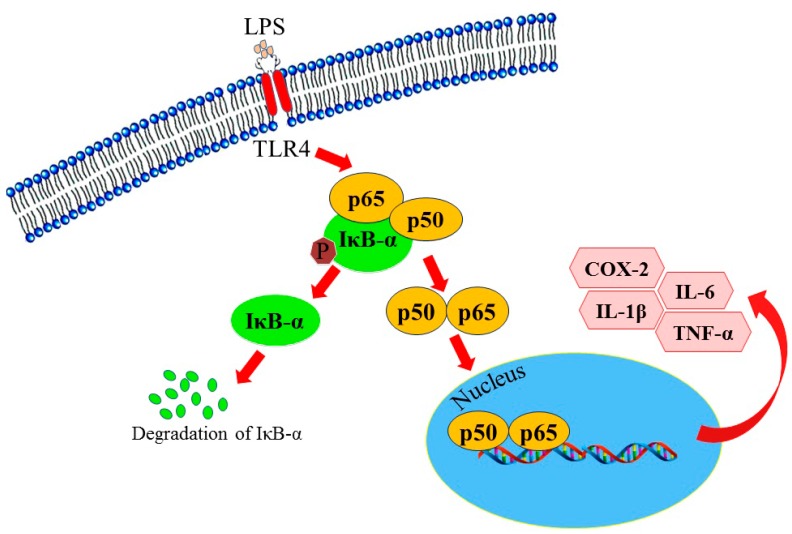
TLR4-mediated NF-κB signaling pathway.

Inflammation is one of the pathologic processes that contribute to disorders. It involves complex signal transduction pathways and the production of various pro-inflammatory factors. Mammalian toll-like receptors (TLRs) are transmembrane receptors that recognize microbial infection and trigger innate immune responses [34]. TLR4 is primarily expressed in macrophages. Binding of lipopolysaccharide (LPS) to TRL4 initiates several signal transduction pathways, including NF-κB [35]. NF-κB is a heterodimer consisting of p65 and a p52 or p50. In cytoplasm, inactivated NF-κB binds with IκB-α. Phosphorylation of IκB-α results in the dissociation of NF-κB from IκB-α, allowing the translocation of heterodimer into the nucleus and binding to the promoters of pro-inflammatory genes, such as interleukine (IL)-1β, IL-6, tumor necrosis factor (TNF)-α, and cyclooxygenase (COX)-2 (Figure 6).

**Figure 7:**
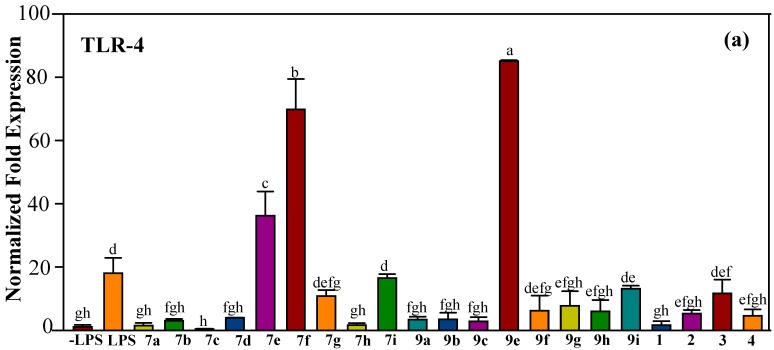

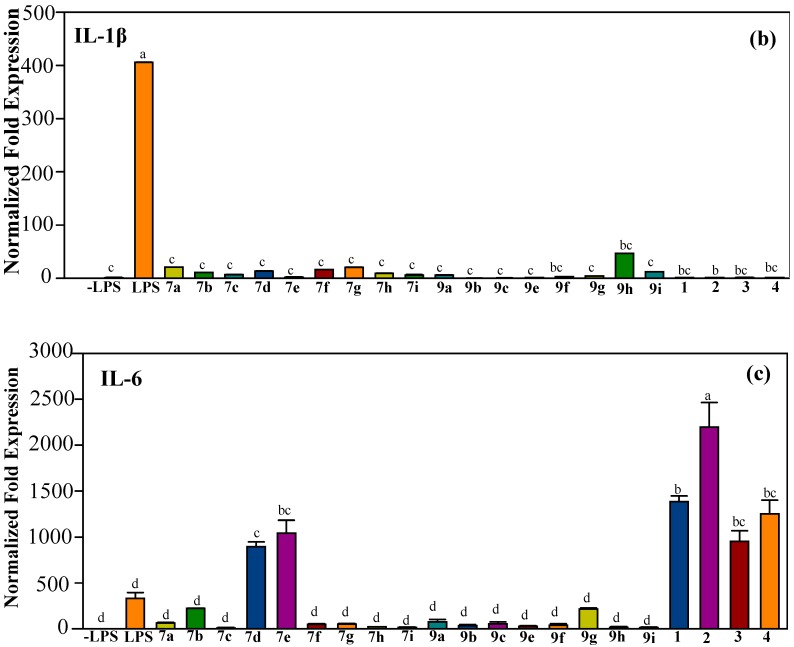
mRNA expression of (**a**) TLR-4, (**b**) IL-1β, and (**c**) IL-6. RAW 264.7 cells were pre-treated with synthetic or natural compounds (10 μg/mL) for 1 h before induction of inflammation by LPS (1 μg/mL) for 6 h. Values indicate means ± SD. Bars with the same letters are not significantly different (α = 0.05). LPS: lipopolysaccharide; **7a**–**i** are synthetic halo-substituted chalcones; **9a**–**i** are synthetic halo-substituted azachalcones; **1**–**4** are synthetic natural products.

**Figure 8:**
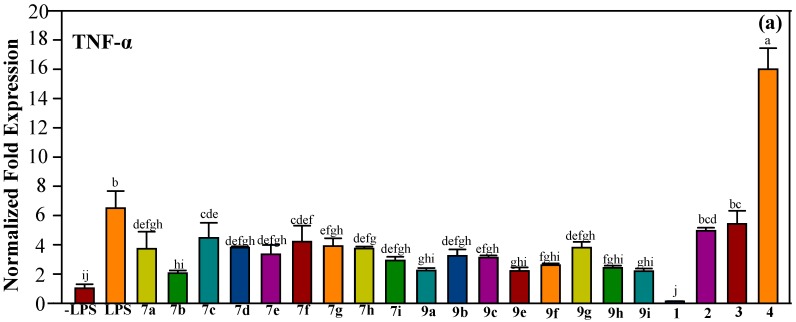

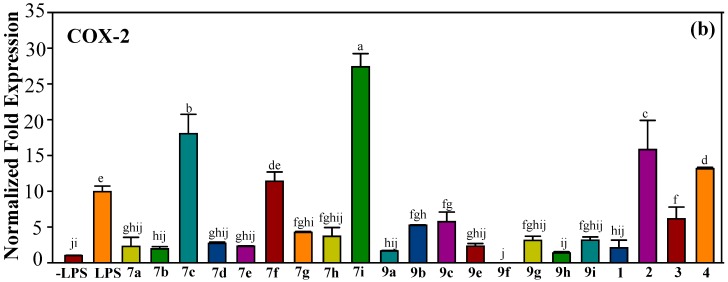
mRNA expression of (**a**) TNF-α and (**b**) COX-2. RAW 264.7 cells were pre-treated with synthetic or natural compounds (10 μg/mL) for 1 h before induction of inflammation by LPS (1 μg/mL) for 6 h. Values indicate means ± SD. Bars with the same letters are not significantly different (α = 0.05). LPS: lipopolysaccharide; **7a**–**i** are synthetic halo-substituted chalcones; **9a**–**i** are synthetic halo-substituted azachalcones; **1**–**4** are synthetic natural products.

Pre-treatment with some synthetic chalcones and azachalcones ameliorated the expression of TLR-4, IL-1β, IL-6, TNF-α, and COX-2 induced by LPS (Figure 7 and Figure 8). IL-1β, IL-6, and TNF-α are pro-inflammatory cytokines secreted by macrophages to initiate and regulate the progression of inflammation [36]. IL-1β is the master inflammatory cytokine in the IL-1 family produced at the early stages of the immune response. IL-1β plays a crucial role in recruitment of monocytes to the inflammation site and interactions between immune cells and nerve cells [37]. Similar to IL-1β, IL-6 is an endogenous pyrogen that promotes fever and the production of acute phase proteins from liver. IL-6 also involves in the recruitment of immune cells to inflammation site [38]. TNF-α was first described for its ability to induce necrosis of tumor cells. Binding of TNF-α to receptors TNFR1 or TNFR2 signals NF-κB activation. Transcriptional induction of TNF-α by NF-κB further amplifies the signaling pathway [39,40]. COX catalyzes the conversion of fatty acids to prostaglandins and thromboxanes. COX-1 is constitutively expressed in all cells, but the expression of COX-2 is induced by pro-inflammatory cytokines [41]. Blocking of IL-1β, IL-6, TNF-α, and COX-2 has become the targeting therapy for the treatment of inflammatory diseases [42,43,44,45].

**Figure 9:**
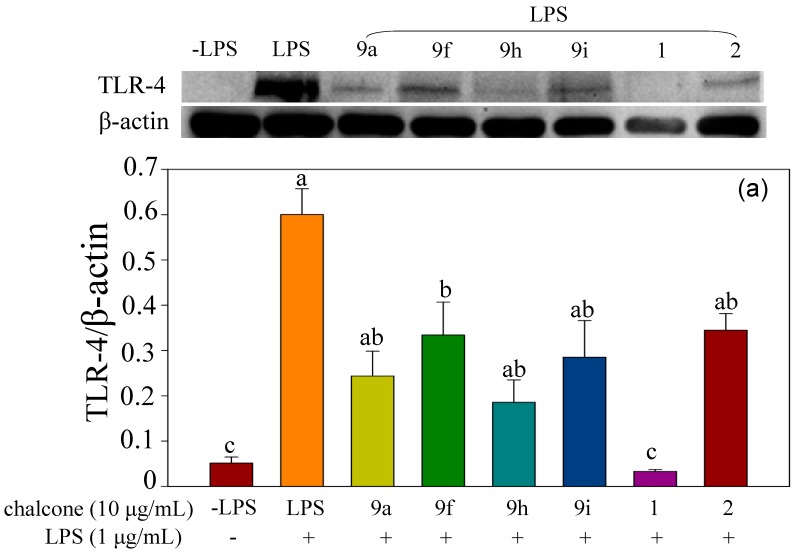

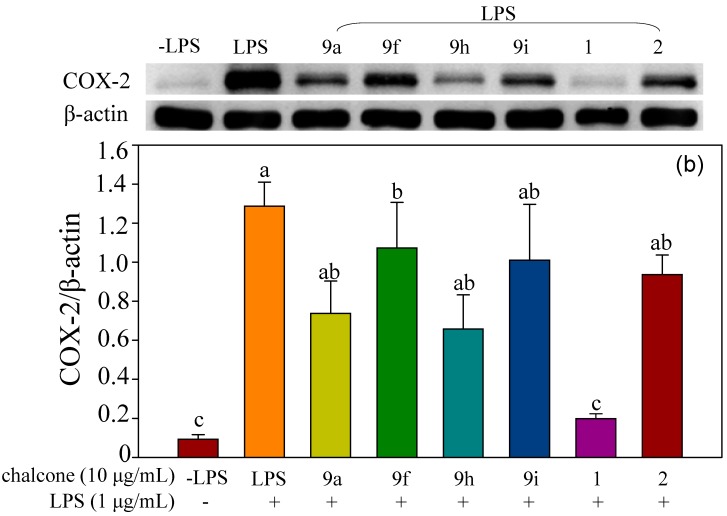
Protein expression of (**a**) TLR4 and (**b**) COX-2. RAW 264.7 cells were pre-treated with synthetic halo-substituted azachalcones **9a**, **9f**, **9h**, **9i** or natural synthetic compounds **1** or **2** (10 μg/mL) for 1 h before induction of inflammation by LPS (1 μg/mL) for 6 h. Values indicate means ± SD. Bars with the same letters are not significantly different (α = 0.05). LPS: lipopolysaccharide.

The anti-inflammatory effects are different among the testing compounds; however, halo-substituted azachalcones **9a**–**i** showed more significant inhibition than halo-substituted chalcones **7a**–**i**. Among the azachalcones, **9a**, **9f**, **9h**, and **9i** showed superior anti-inflammatory activity. Therefore, protein expression of TLR4 and COX-2 was examined in RAW 264.7 cells pre-treated with **9a**, **9f**, **9h**, or **9i** before induction of inflammation. The results in Figure 9 show pre-treatment with these four halo-substituted azachalcones attenuated the inflammatory responses induced by LPS. Compared with the natural chalcones **1** and **2**, these four synthetic compounds showed more significant inhibition on inflammation than compound **2**, but not compound **1**.

It has been reported that the electron-withdrawing groups in B-ring, as well as the electron-donating groups in A-ring on chalcone derivatives, enhanced the cytotoxic activity [30]. Since the C-2 OH group of chalcones involved intramolecular hydrogen bonding with the carbonyl group, however, the C-2 nitrogen atom of azachalcones **9a**–**i**, were to lack this attractive force. Therefore, we suspect that this difference may play a crucial role in their activity.

Based on the slight difference in structures between chalcones and azachalcones and comments on cytotoxic activity of chalcones, we intended to realize the role of hydroxyl and nitrogen atom in A ring of respective molecules. Therefore, we synthesized a series of monohalo substitutes in B ring of both chalcones (**7a**–**i**) and azachalcone (**9a**–**i**). This paper is the first to report the comparison of anti-inflammatory activity between synthetic halo-chalcones and -azachalcones. The results will lay the background for future development of anti-inflammatory nutraceuticals. Evaluation of bioavailability and toxicity of **9a**, **9f**, **9h**, and **9i** halo-azachalcones is taking place in our laboratory to pioneer in vivo functional study.

## 3. Materials and Methods

Chemicals used for organic synthesis were purchased from commercial sources and used without purification except when otherwise stated. ^1^H NMR (600 MHz) and ^13^C NMR (150 MHz) spectra were recorded on a Bruker 600 instrument. Chemical shifts were reported in ppm and referenced to the residue of solvent: (CDCl_3_: 7.26 ppm for ^1^H; 77.0 ppm for ^13^C). Melting points were determined on Fargo MP-2D and not corrected. HRMS were recorded on Finnigan MAT-95S. Dulbecco’s modified Eagle’s medium (DMEM), lipopolysaccharide (LPS), and dimethyl sulfoxide (DMSO) were purchased from Sigma (St. Louis, MO, USA). Fetal bovine serum (FBS) and penicillin-streptomycin were obtained from Biowest (Kancas, MO, USA). Anti-TLR4 (rabbit anti-toll-like receptor 4 polyclonal antibody), anti-COX-2 (rabbit anti-cyclooxygenase-2 polyclonal antibody), and anti-β-actin (mouse anti-beta actin monoclonal antibody) antibodies were purchased from Novus (Littleton, CO, USA). Peroxidase-conjugated sheep anti-mouse IgG and goat anti-rabbit IgG were obtained from Jackson ImmunoResearch (West Grove, PA, USA).

### 3.1. General Procedure of Claisen-Schmidt Condensation in Synthesis of ***7a***–***i***

To a stirred solution of 5 (0.100 mL, 0.108 g, and 0.890 mmol), for example, EtOH (2.5 mL) was added the three equivalents of 8M KOH. This mixture was stirred at 0 °C for 10 min, followed by addition of the corresponding aldehyde (1.5 equiv.) at that temperature. The mixture was gradually warmed up to room temperature until **5** was consumed by TLC detection. At the end of reaction time, the mixture was neutralized with 2N HCl, diluted with H_2_O, and extracted with CH_2_Cl_2_. The organic layer was dried over MgSO_4_ and purified by flash column chromatography.

#### 3.1.1. *(E)-3-(4-Fluorophenyl)-1-(2-hydroxyphenyl)prop-2-en-1-one* (**7a**)

Purification by flash column chromatography (CH_2_Cl_2_:Hexanes = 1:5–1:3). m.p. 118–120 °C (lit. [29] 120–122 °C). Yellow solid. ^1^H NMR (600 MHz, CDCl_3_) *δ* 12.79 (s, 1H), 7.90 (d, *J* = 8.0 Hz, 1H), 7.88 (d, *J* = 15.5 Hz, 1H), 7.66 (d, *J* = 5.6 Hz, 1H), 7.65 (d, *J* = 5.6 Hz, 1H), 7.58 (d, *J* = 15.5 Hz, 1H), 7.50 (t, *J* = 8.2 Hz, 1H), 7.13 (d, *J* = 8.5 Hz, 1H), 7.12 (d, *J* = 8.5 Hz, 1H), 7.03 (d, *J* = 8.5 Hz, 1H), 6.94 (t, *J* = 7.4 Hz, 1H). ^13^C NMR (150 MHz, CDCl_3_) *δ* 193.5, 164.2 (^1^*J* = 251 Hz), 163.6, 140.1, 136.4, 130.8 (^4^*J* = 1.5 Hz), 130.6 (^3^*J* = 7.5 Hz) (×2), 129.6, 119.9, 119.8, 118.9, 118.6, 116.3, 116.2 (^2^*J* = 22.5 Hz) (×2). HRMS (ESI) calculated for C_15_H_12_FO_2_ [M + H]^+^ 243.0821. Found: 243.0823.

#### 3.1.2. *(E)-3-(4-Chlorophenyl)-1-(2-hydroxyphenyl)prop-2-en-1-one* (**7b**)

Purification by flash column chromatography (CH_2_Cl_2_:Hexanes = 1:5–1:3). m.p. 152–154 °C (lit. [29] 143–145 °C). Yellow solid. ^1^H NMR (600 MHz, CDCl_3_) *δ* 12.75 (s, 1H), 8.00 (d, *J* = 15.7 Hz, 1H), 7.92 (dd, *J* = 8.2, 1.7 Hz, 1H), 7.79 (d, *J* = 15.7 Hz, 1H), 7.66 (td, *J* = 7.6, 1.7 Hz, 1H), 7.51 (ddd, *J* = 8.5, 7.2, 1.6 Hz, 1H), 7.44–7.39 (m, 1H), 7.22 (td, *J* = 7.6, 1.0 Hz, 1H), 7.16 (ddd, *J* = 9.2, 8.2, 0.9 Hz, 1H), 7.04 (dd, *J* = 8.5, 1.0 Hz, 1H), 6.96 (td, *J* = 7.5, 1.1 Hz, 1H). ^13^C NMR (150 MHz, CDCl_3_) *δ* 193.8, 163.7, 162.8, 161.1, 138.3, 136.5, 132.2, 130.2, 129.8, 124.6, 122.9, 120.0, 118.9, 118.7, 116.4. HRMS (ESI) calculated for C_15_H_12_ClO_2_ [M]^+^ 259.0526. Found: 259.0530.

#### 3.1.3. *(E)-3-(4-Bromophenyl)-1-(2-hydroxyphenyl)prop-2-en-1-one* (**7c**)

Purification by flash column chromatography (CH_2_Cl_2_:Hexanes = 1:3–1:2). m.p. 145–148 °C. (lit. [29] 150–152 °C). Yellow solid. ^1^H NMR (600 MHz, CDCl_3_) *δ* 12.73 (s, 1H), 7.91 (d, *J* = 8.0 Hz, 1H), 7.85 (d, *J* = 15.5 Hz, 1H), 7.65 (d, *J* = 15.5 Hz, 1H), 7.58 (d, *J* = 8.3 Hz, 2H), 7.55–7.49 (m, 3H), 7.04 (d, *J* = 8.3 Hz, 1H), 6.95 (t, *J* = 7.3 Hz, 1H). ^13^C NMR (150 MHz, CDCl_3_) 193.5, 163.6, 144.0, 136.6, 133.5, 132.3, 130.0 (x3), 129.6, 125.3, 120.7, 119.9, 118.9, 118.7. HRMS (ESI) calculated for C_15_H_12_BrO_2_ [M + H]^+^ 303.0021. Found: 303.0025.

#### 3.1.4. *(E)-3-(3-Fluorophenyl)-1-(2-hydroxyphenyl)prop-2-en-1-one* (**7d**)

Purification by flash column chromatography (CH_2_Cl_2_:Hexanes = 1:8–1:6). m.p. 100–101 °C (lit. [29] 109–110 °C). Yellow solid. ^1^H NMR (600 MHz, CDCl_3_) *δ* 12.70 (s, 1H), 7.91 (d, *J* = 8.1 Hz, 1H), 7.86 (d, *J* = 15.4 Hz, 1H), 7.64 (d, *J* = 15.4 Hz, 1H), 7.52 (t, *J* = 7.7 Hz, 1H), 7.44–7.40 (m, 2H), 7.36 (d, *J* = 9.6 Hz, 1H), 7.14 (t, *J* = 7.8 Hz, 1H), 7.04 (t, *J* = 8.3 Hz, 1H), 6.96 (t, *J* = 7.4 Hz, 1H). ^13^C NMR (150 MHz, CDCl_3_) *δ* 193.4, 163.6, 163.0 (^1^*J* = 246 Hz), 143.9, 136.9, 136.7 (^2^*J* = 33 Hz), 130.6 (^3^*J* = 9 Hz), 129.6, 124.7, 121.4, 119.9 (×3), 118.8 (^2^*J* = 36 Hz), 117.7 (^2^*J* = 21 Hz), 114.6 (^2^*J* = 21 Hz). HRMS (ESI) calculated for C_15_H_12_FO_2_ [M + H]^+^ 243.0821. Found: 243.0825.

#### 3.1.5. *(E)-3-(3-Chlorophenyl)-1-(2-hydroxyphenyl)prop-2-en-1-one* (**7e**)

Purification by flash column chromatography (CH_2_Cl_2_:Hexanes = 1:5–1:3). m.p. 106–108 °C (lit. [29] 105–107 °C). Yellow-orange solid.^1^H NMR (600 MHz, CDCl_3_) *δ* 12.71 (s, 1H), 7.91 (d, *J* = 8.0 Hz, 1H), 7.83 (d, *J* = 15.5 Hz, 1H), 7.65 (s, 1H), 7.63 (d, *J* = 15.1 Hz, 1H), 7.52–7.49 (m, 2H), 7.40 (t, *J* = 7.9 Hz, 1H), 7.37 (t, *J* = 7.9 Hz, 1H), 7.03 (d, *J* = 8.4 Hz, 1H), 6.96 (t, *J* = 7.6 Hz, 1H). ^13^C NMR (150 MHz, CDCl_3_) *δ* 193.4, 163.6, 143.6, 136.6, 136.4, 135.1, 130.7, 130.3, 129.7, 128.0, 127.0, 121.5, 119.9, 118.9, 118.7. HRMS (ESI) calculated for C_15_H_12_ClO_2_ [M]^+^ 259.0526. Found: 259.0523.

#### 3.1.6. *(E)-3-(3-Bromophenyl)-1-(2-hydroxyphenyl)prop-2-en-1-one* (**7f**)

Purification by flash column chromatography (CH_2_Cl_2_:Hexanes = 1:5–1:3). m.p. 98–100 °C (lit. [29] 108–110 °C). Yellow solid. ^1^H NMR (600 MHz, CDCl_3_) *δ* 12.70 (s, 1H), 7.92 (d, *J* = 8.0 Hz, 1H), 7.83 (d, *J* = 15.5 Hz, 1H), 7.82 (s, 1H), 7.64 (d, *J* = 15.5 Hz, 1H), 7.56 (d, *J* = 7.6 Hz, 2H), 7.52 (t, *J* = 7.5 Hz, 1H), 7.32 (t, *J* = 7.9 Hz, 1H), 7.04 (d, *J* = 8.3 Hz, 1H), 6.96 (t, *J* = 7.4 Hz, 1H). ^13^C NMR (150 MHz, CDCl_3_) *δ* 193.4, 163.7, 143.6, 136.7 (x2), 133.6, 131.0, 130.5, 129.7, 127.5, 123.2, 121.5, 119.9, 119.0, 118.7. HRMS (ESI) calculated for C_15_H_12_BrO_2_ [M + H]^+^ 303.0021. Found: 303.0023.

#### 3.1.7. *(E)-3-(2-Fluorophenyl)-1-(2-hydroxyphenyl)prop-2-en-1-one* (**7g**)

Purification by flash column chromatography (CH_2_Cl_2_:Hexanes = 1:4–1:3). m.p. 90–93 °C (lit. [46] 80 °C). Yellow solid. ^1^H NMR (600 MHz, CDCl_3_) *δ* 12.76 (s, 1H), 7.91 (d, *J* = 8.1 Hz, 1H), 7.78 (d, *J* = 15.7 Hz, 1H), 7.65 (t, *J* = 7.5 Hz, 1H), 7.51 (t, *J* = 7.3 Hz, 1H), 7.41 (dd, *J* = 13.6, 6.4 Hz, 1H), 7.22 (t, *J* = 7.6 Hz, 1H), 7.15 (dd, *J* = 10.6, 8.6 Hz, 1H), 7.03 (d, *J* = 8.3 Hz, 1H), 6.95 (t, *J* = 7.6 Hz, 1H). ^13^C NMR (150 MHz, CDCl_3_) *δ* 193.8, 163.6, 161.9 (^1^*J* = 254.0 Hz), 138.2, 136.5, 132.2 (^3^*J* = 8.8 Hz), 130.1, 129.7, 124.5 (^4^*J* = 3.0 Hz), 122.8 (^3^*J* = 7.9 Hz), 122.7, 120.0, 118.9, 118.6, 116.4 (^2^*J* = 23.0 Hz). HRMS (ESI) calculated for C_15_H_12_FO_2_ [M + H]^+^ 242.0821. Found: 243.0825.

#### 3.1.8. *(E)-3-(2-Chlorophenyl)-1-(2-hydroxyphenyl)prop-2-en-1-one* (**7h**)

Purification by flash column chromatography (CH_2_Cl_2_:Hexanes = 1:3–1:2). m.p. 103–105 °C (lit. [47] 138–140 °C).Yellow solid. ^1^H NMR (600 MHz, CDCl_3_) *δ* 12.72 (s, 1H), 8.31 (d, *J* = 15.6 Hz, 1H), 7.91 (d, *J* = 8.0 Hz, 1H), 7.77 (dd, *J* = 7.6, 1.3 Hz, 1H), 7.65 (d, *J* = 15.5 Hz, 1H), 7.52 (t, *J* = 7.3 Hz, 1H), 7.47 (d, *J* = 7.7 Hz, 1H), 7.36 (td, *J* = 8.1, 2.1 Hz, 1H), 7.34 (t, *J* = 6.9 Hz, 1H), 7.04 (d, *J* = 8.3 Hz, 1H), 6.95 (t, *J* = 7.9 Hz, 1H). ^13^C NMR (150 MHz, CDCl_3_) *δ* 193.6, 163.7, 141.2, 136.5, 135.8, 133.0, 131.5, 130.4, 129.7, 128.0, 127.1, 122.9, 119.9, 118.9, 118.7. HRMS (ESI) calculated for C_15_H_12_ClO_2_ [M + H]^+^ 259.0526. Found: 259.0527.

#### 3.1.9. *(E)-3-(2-Bromophenyl)-1-(2-hydroxyphenyl)prop-2-en-1-one* (**7i**)

Purification by flash column chromatography (CH_2_Cl_2_:Hexanes = 1:4–1:3). m.p. 103–105 °C (lit. [48] 103–104 °C). Yellow solid. ^1^H NMR (600 MHz, CDCl_3_) *δ* 12.71 (s, 1H), 8.26 (d, *J* = 15.6 Hz, 1H), 7.90 (d, *J* = 8.0 Hz, 1H), 7.75 (d, *J* = 7.8 Hz, 1H), 7.66 (d, *J* = 8.0 Hz, 1H), 7.59 (d, *J* = 15.4 Hz, 1H), 7.51 (td, *J* = 8.3, 1.1 Hz, 1H), 7.38 (t, *J* = 7.4 Hz, 1H), 7.27 (td, *J* = 7.6, 1.6 Hz, 1H), 7.04 (d, *J* = 8.3 Hz, 1H), 6.95 (t, *J* = 7.3 Hz, 1H). ^13^C NMR (150 MHz, CDCl_3_) *δ* 193.6, 163.6, 143.7, 136.6, 134.8, 133.7, 131.6, 129.7, 127.8, 126.1, 123.0, 119.9, 118.9, 118.7. HRMS (ESI) calculated for C_15_H_12_BrO_2_ [M + H]^+^ 303.0021. Found: 303.0019.

### 3.2. Biological Evaluation

#### 3.2.1. Cell Culture

Mouse BALB/c macrophage cell line RAW 264.7, which was obtained from Biosource Collection and Research Center of Food Industry Research and Development Institute (Shinchu, Taiwan), was cultured in DMEM supplemented with FBS (10%), sodium bicarbonate (0.22%), streptomycin (100 units/mL), and penicillin (100 units/mL) in a 37 °C incubator with 5% CO_2_. When 50% confluence was reached, the cells were cultured with various synthetic chalcones for 1 h before LPS was added to the medium (final concentration 1 μg/mL) to induce inflammation. Six hours later, the cells were harvested for real-time PCR analysis and immunoblot analysis [49].

#### 3.2.2. Cell Viability Assay

RAW 264.7 cells were seeded in 96-well plate at a concentration of 3 × 10^5^/well. Twenty-four hours after seeding, the cells were treated with various concentrations of synthetic chalcones and LPS (1 μg/mL) for another 24 h before the medium was removed and the cells were cultured with new medium containing MTT (3-(4,5-dimethylthiazol-2-yl)-2,5-diphenyltetrazolium bromide) at a final concentration of 0.5 mg/mL. Four hours later, the purple formazan was dissolved by DMSO, and the absorbance at 570 nm was taken. The absorbance is proportional to the viability of cells [49].

#### 3.2.3. Quantitative Real-Time PCR Analysis

Effects of synthetic chalcones on the expressions of following genes were quantified: signal recognition particle 72 (SRP 72), IκB-α, cycloxygenase-2 (COX-2), interleukin-6 (IL-6), interleukin-1β (IL-1β), tumor necrosis factor-α(TNF-α), and inducible nitric oxide synthase (iNOS). Total RNA was extracted from the cells by using Aurum™ Total RNA MiniKit (BIO-RAD, Hercules, CA, USA). Complementary DNA (cDNA) was synthesized by using iScript™ cDNA Synthesis Kit (BIO-RAD, Hercules, CA, USA) and performed on T3 Thermocycler (Biometra, Germany) with the program of 25 °C for 5 min, 42 °C for 30 min, and 85 °C for 5 min. The cDNA (5 ng/μL) was mixed with SYBR green (iQ™ SYBR Green Supermix, BIO-RAD, Hercules, CA, USA) and primers (Table 3). The mixture was subject to the thermal cycling under the following conditions: heating up to 95 °C in 3 min, followed by 40 cycles at 95 °C for 10 s and 50 °C for 30 s. The expression of each gene was normalized to the corresponding SRP 72 (internal reference) threshold cycle (ct) values by the 2^−ΔΔct^ method [50].

#### 3.2.4. Immunoblot Analysis

Cells were harvested and resuspended in lysis buffer containing 50 mM Tris-HCl, 1% Noniswr P40, and 150 mM sodium chloride before centrifuged at 10,000× *g* for 30 min. Proteins in supernatant were separated by sodium dodecyl sulfate polyacrylamide gel electrophoresis (SDS-PAGE) and transferred to polyvinylidene fluoride (PVDF) membrane (PerkinElmer, Waltham, MA, USA). Five percent stacking gel and 8% separating gel were used for SDS-PAGE. The membranes were blocked with 5% nonfat dry milk in Tris-buffered saline containing Tween (TBST) (20 mM Tris-HCl, 137 mM NaCl, 0.1% Tween-20, pH 8.3) for one hour before being incubated with primary antibody (anti-TLR4, anti-COX-2, or anti-β-actin) overnight at 4 °C and horseradish peroxidase-conjugated secondary antibody for 1 h at room temperature. An enhanced chemiluminescence kit (ECL, PerkinElmer, Waltham, MA, USA) was applied to detect the immunoreactive proteins [49].

## 4. Conclusions

A series of monohalo-substituted chalcones and azachalcones were synthesized and compared their anti-inflammatory activity with a concentration of 10 μg/mL. In this series, azachalcones exerted more significant activity than chalcones on inhibition of pro-inflammatory factor expression, including TLR4, IκB-α, p65, IL-1β, IL-6, TNF-α, and COX-2. In lack of the internal hydrogen bonding between C-2 hydroxy group and carbonyl group as in chalcones, our results indicate that the nitrogen atom in azachalcones might be important to contribute their activity. Therefore, azachalcones **9a**, **9f**, **9h,** and **9i** used for further investigation is undergoing in our laboratory.

## Figures and Tables

**Table 1:** Optimization in synthesis of chalcones **7a**–**i**.

Entry	Compound	Time (h)	Yield%
1	**7a**	14	92
2	**7b**	9	75
3	**7c**	15	82
4	**7d**	8	76
5	**7e**	13	67
6	**7f**	14	73
7	**7g**	13	85
8	**7h**	14	78
9	**7i**	15	73

Condition: **5** (1 equiv.), **6a**–**i** (1.5 equiv.), KOH (3 equiv.), EtOH (0.2 M), and 0 to r.t.

**Table 2:** Cell viability of RAW 264.7 cells after treated with various concentrations of halo-substituted chalcones **7a**–**i**, azachalcones **9a**–**i**, or synthetic natural products **1**–**4** for 24 h.

Compound	Concentration (μg/mL)				
	0	10	25	50	100
**7a**	100.0	114.27 ± 4.83 ^1^	99.76 ± 18.08	95.02 ± 21.09	80.32 ± 15.54
**7b**	100.0	83.55 ± 12.07	105.08 ± 14.37	90.34 ± 19.48	55.51 ± 4.21
**7c**	100.0	110.86 ± 29.05	115.31 ± 15.25	79.03 ± 6.30	65.77 ± 12.03
**7d**	100.0	97.37 ± 20.37	86.35 ± 18.47	71.78 ± 10.27	34.72 ± 4.15
**7e**	100.0	83.42 ± 13.10	69.68 ± 5.17	45.95 ± 6.08	17.24 ± 1.29
**7f**	100.0	71.29 ± 8.12	56.92 ± 4.88	30.60 ± 4.96	19.32 ± 2.89
**7g**	100.0	80.47 ± 8.38	75.60 ± 12.85	68.21 ± 7.45	42.19 ± 4.87
**7h**	100.0	101.07 ± 8.63	87.16 ± 9.03	60.71 ± 7.09	16.35 ± 0.56
**7i**	100.0	87.00 ± 18.69	64.16 ± 6.38	32.19 ± 3.32	24.23 ± 2.00
**9a**	100.0	99.00 ± 3.19	93.66 ± 3.87	70.68 ± 2.17	4.22 ± 0.47
**9b**	100.0	123.61 ± 0.10	112.57 ± 8.72	82.18 ± 3.87	3.92 ± 0.19
**9c**	100.0	93.28 ± 9.09	69.54 ± 23.80	14.51 ± 2.05	5.52 ± 0.37
**9e**	100.0	90.39 ± 10.15	85.06 ± 8.59	63.56 ± 3.03	8.60 ± 1.06
**9f**	100.0	102.57 ± 16.87	71.97 ± 8.07	50.11 ± 9.90	15.27 ± 1.64
**9g**	100.0	86.56 ± 12.35	89.25 ± 5.68	34.25 ± 3.08	3.3 ± 0.22
**9h**	100.0	84.49 ± 8.66	86.32 ± 8.10	60.61 ± 25.05	43.90 ± 18.91
**9i**	100.0	79.14 ± 11.53	77.95 ± 10.43	67.49 ± 8.26	33.68 ± 5.03
	**0**	**1**	**5**	**10**	**25**
**1**	100.0	105.09 ± 12.45	103.04 ± 5.41	75.05 ± 7.95	23.64 ± 4.82
**2**	100.0	99.80 ± 14.60	102.52 ± 10.87	87.14 ± 14.13	51.99 ± 6.98
**3**	100.0	86.98 ± 6.15	81.19 ± 8.43	64.09 ± 4.80	34.01 ± 4.21
**4**	100.0	90.63 ± 16.23	103.44 ± 8.52	85.54 ± 12.25	45.88 ± 6.48

^1^ The results are presented as % of cell number of control group.

**Table 3:** Forward and reverse primers.

Gene	Forward Primer 5′3′	Reverse Primer 5′3′
*SRP72*	CACACCCTAGCCCAACTTATT	TCAAGCGCCTCAACATCTAC
*TLR4*	TTCAGAACTTCAGTGGCTGGATTTA	GTCTCCACAGCCACCAGATTCTC
*IκB-α*	CCTTCCTCAACTTCCAGAACAA	GATCACAGCCAGCTTTCAGA
*p65*	TGTGGAGATCATCGAACAGCCGAA	TGTTCCTGGTCCTGTGTAGCCATT
*IL-1β*	GTTACATCAGCACCTCACAA	TTAGAAACAGCTCAGCCCATAC
*IL-6*	CTTCCATCCAGTTGCCTTCT	CTCCGACTTGTGAAGTGGTATAG
*TNF-α*	TTGTCTACTCCCAGGTTCTCT	GAGGTTGACTTTCTCCTGGTATG
*COX-2*	CGGACTGGATTCTATGGTGAAA	CTTGAAGTGGGTCAGGATGTAG

## Supplementary Materials

The following are available online. The ^1^H and ^13^C NMR data for compounds **7a**–**i** and **9a**–**i****.**
