# Supplementary material for: Halo-Substituted Chalcones and Azachalcones Inhibited Lipopolysaccharited-Stimulated Pro-Inflammatory Responses through the TLR4-Mediated Pathway
Source: Molecules. 2018 Mar 7;23(3):597. doi: 10.3390/molecules23030597 (PMC6017711; doi:10.3390/molecules23030597)
Supplement: Supplementary file 1 [file molecules-23-00597-s001.pdf]

Supplementary materials

Halo-substituted Chalcones and Azachalcones Inhibited Lipopolysaccharited-Stimulated Pro-Inflammatory Responses through TLR4-mediated Pathway

Tzenge-Lien Shih[a], Ming-Hwa Liu[b], Chia-Wai Li[a], Chia-Feng Kuo[b*]

[a] *Department of Chemistry, Tamkang University, Tamsui Dist., New Taipei City, Taiwan*
[b]*Department of Food Science, Nutrition, and Nutraceutical Biotechnology, Shih Chien University, Zhongshan Dist., Taipei, Taiwan*

[*]Corresponding authors. Chia-Feng Kuo: 70, Ta-Chih Street, Taipei, Taiwan. Tel: 886-2-25381111 ext. 6214; Fax: 886-2-25334789; E-mail: drkuo@mail.usc.edu.tw.

^{1}H NMR (600 MHz, $CDCl_3$) of Compound **7a** ········ S3
^{13}C NMR (150 MHz, $CDCl_3$) of Compound **7a** ········ S4
^{1}H NMR (600 MHz, $CDCl_3$) of Compound **7b** ········ S5
^{13}C NMR (150 MHz, $CDCl_3$) of Compound **7b** ········ S6
^{1}H NMR (600 MHz, $CDCl_3$) of Compound **7c** ········ S7
^{13}C NMR (150 MHz, $CDCl_3$) of Compound **7c** ········ S8
^{1}H NMR (600 MHz, $CDCl_3$) of Compound **7d** ········ S9
^{13}C NMR (150 MHz, $CDCl_3$) of Compound **7d** ········ S10
^{1}H NMR (600 MHz, $CDCl_3$) of Compound **7e** ········ S11
^{13}C NMR (150 MHz, $CDCl_3$) of Compound **7e** ········ S12
^{1}H NMR (600 MHz, $CDCl_3$) of Compound **7f** ········ S13
^{13}C NMR (150 MHz, $CDCl_3$) of Compound **7f** ········ S14
^{1}H NMR (600 MHz, $CDCl_3$) of Compound **7g** ········ S15
^{13}C NMR (150 MHz, $CDCl_3$) of Compound **7g** ········ S16
^{1}H NMR (600 MHz, $CDCl_3$) of Compound **7h** ········ S17
^{13}C NMR (150 MHz, $CDCl_3$) of Compound **7h** ········ S18
^{1}H NMR (600 MHz, $CDCl_3$) of Compound **7i** ········ S19
^{13}C NMR (150 MHz, $CDCl_3$) of Compound **7i** ········ S20

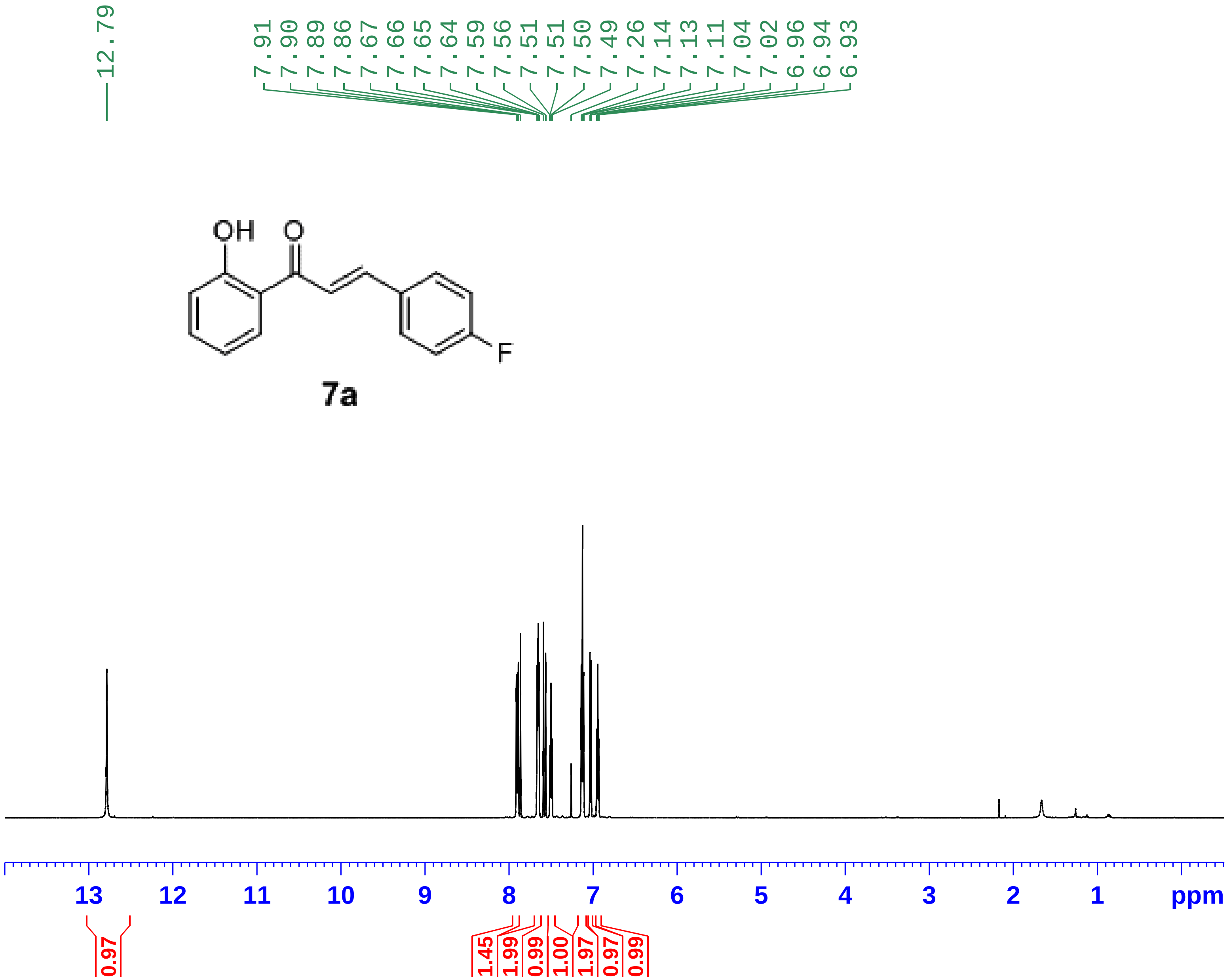

Current Data Parameters
NAME LCW2-100
EXPNO 1
PROCNO 1

F2 - Acquisition Parameters
Date_ 20150504
Time 8.40
INSTRUM spect
PROBHD 5 mm TXI 1H/D-
PULPROG zg30
TD 32768
SOLVENT CDCl3
NS 16
DS 0
SWH 8992.806 Hz
FIDRES 0.274439 Hz
AQ 1.8219008 sec
RG 90.5
DW 55.600 usec
DE 6.00 usec
TE 300.0 K
D1 2.00000000 sec
TD0 1

======== CHANNEL f1 ========
NUC1 1H
P1 9.50 usec
PL1 -3.30 dB
PL1W 42.96254349 W
SFO1 600.1342009 MHz

F2 - Processing parameters
SI 16384
SF 600.1300101 MHz
WDW no
SSB 0
LB 0 Hz
GB 0
PC 1.00

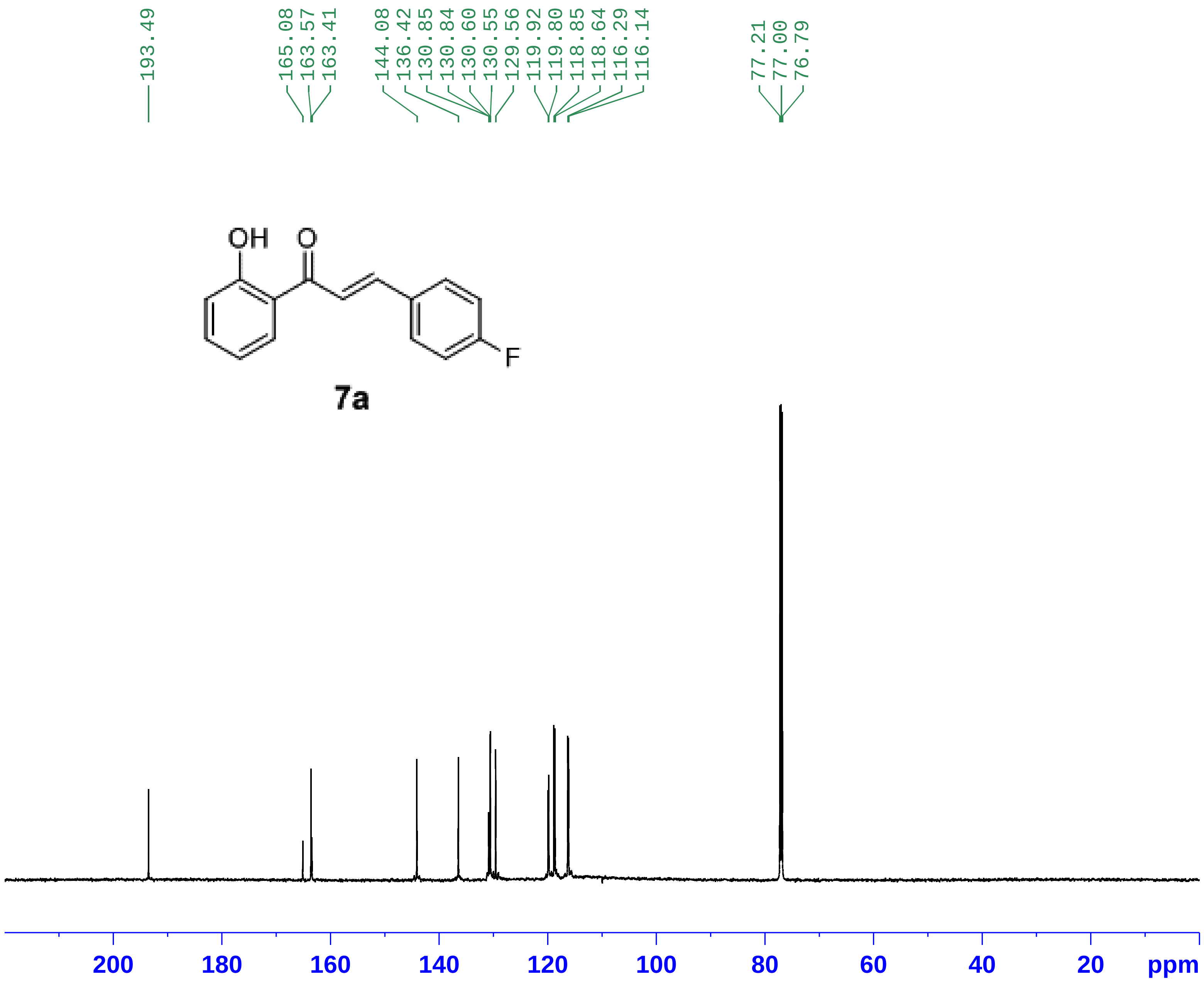

7a

Current Data Parameters
NAME LCW2-100
EXPNO 2
PROCNO 1

F2 - Acquisition Parameters
Date_ 20150504
Time 8.47
INSTRUM spect
PROBHD 5 mm TXI 1H/D-
PULPROG zgpg30
TD 65536
SOLVENT CDCl3
NS 1124
DS 0
SWH 36231.883 Hz
FIDRES 0.552855 Hz
AQ 0.9043968 sec
RG 7290
DW 13.800 usec
DE 6.00 usec
TE 300.0 K
D1 2.40000010 sec
D11 0.03000000 sec
TD0 1

======== CHANNEL f1 ========
NUC1 13C
P1 10.95 usec
PL1 -4.00 dB
PL1W 236.60900879 W
SFO1 150.9194083 MHz

======== CHANNEL f2 ========
CPDPRG[2 waltz16
NUC2 1H
PCPD2 90.00 usec
PL2 2.00 dB
PL12 19.00 dB
PL13 21.00 dB
PL2W 12.67914581 W
PL12W 0.25298220 W
PL13W 0.15962099 W
SFO2 600.1339008 MHz

F2 - Processing parameters
SI 32768
SF 150.9028148 MHz
WDW EM
SSB 0
LB 3.00 Hz
GB 0
PC 1.00

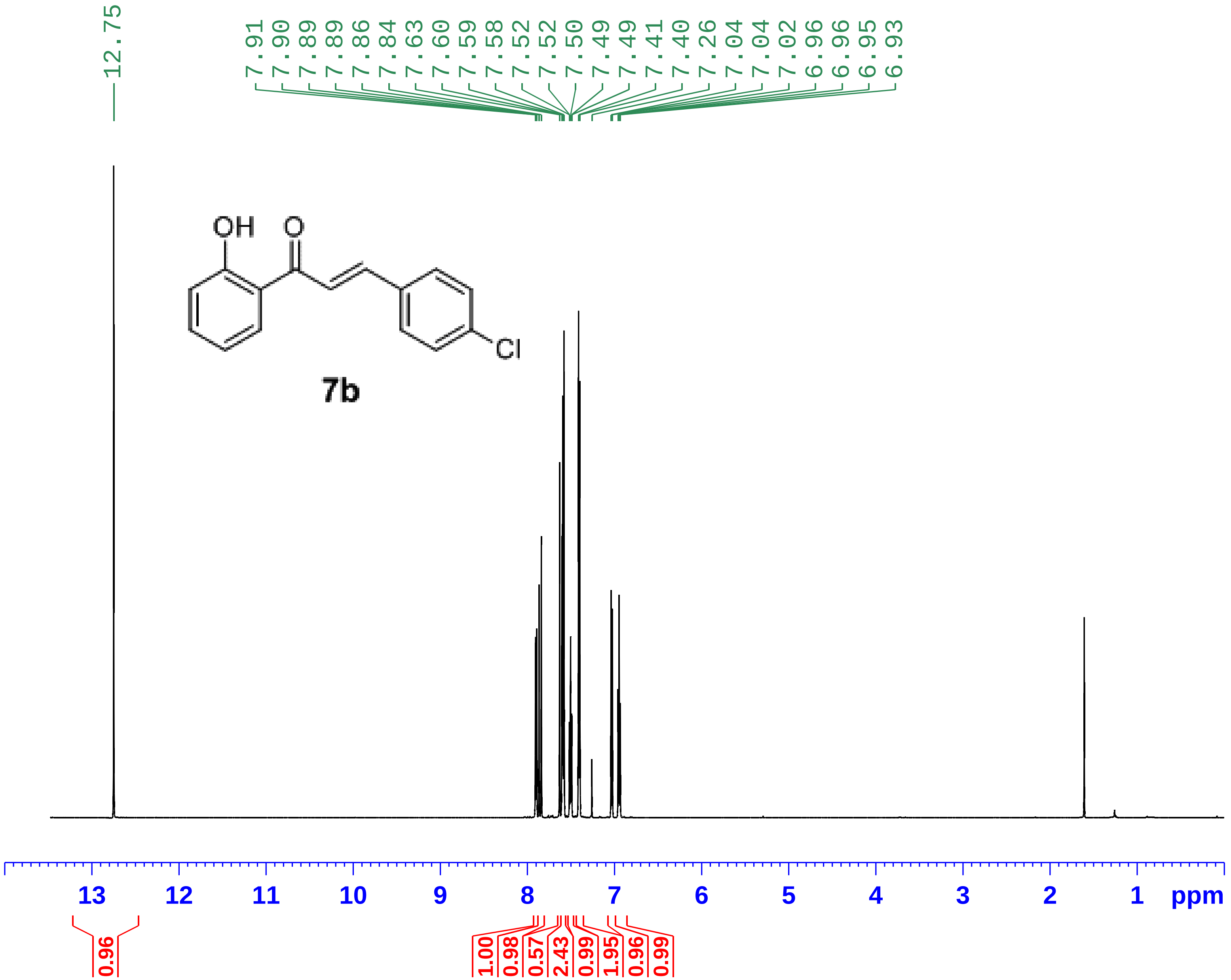

Current Data Parameters
NAME STH1-83
EXPNO 1
PROCNO 1

F2 - Acquisition Parameters
Date_ 20170607
Time 11.34
INSTRUM spect
PROBHD 5 mm TXI 1H/D-
PULPROG zg30
TD 32768
SOLVENT CDCl3
NS 16
DS 0
SWH 8992.806 Hz
FIDRES 0.274439 Hz
AQ 1.8219008 sec
RG 101
DW 55.600 usec
DE 6.00 usec
TE 300.0 K
D1 2.00000000 sec
TD0 1

======== CHANNEL f1 ========
NUC1 1H
P1 8.50 usec
PL1 -1.80 dB
PL1W 30.41515160 W
SFO1 600.1336008 MHz

F2 - Processing parameters
SI 16384
SF 600.1300100 MHz
WDW EM
SSB 0
LB 0 Hz
GB 0
PC 1.00

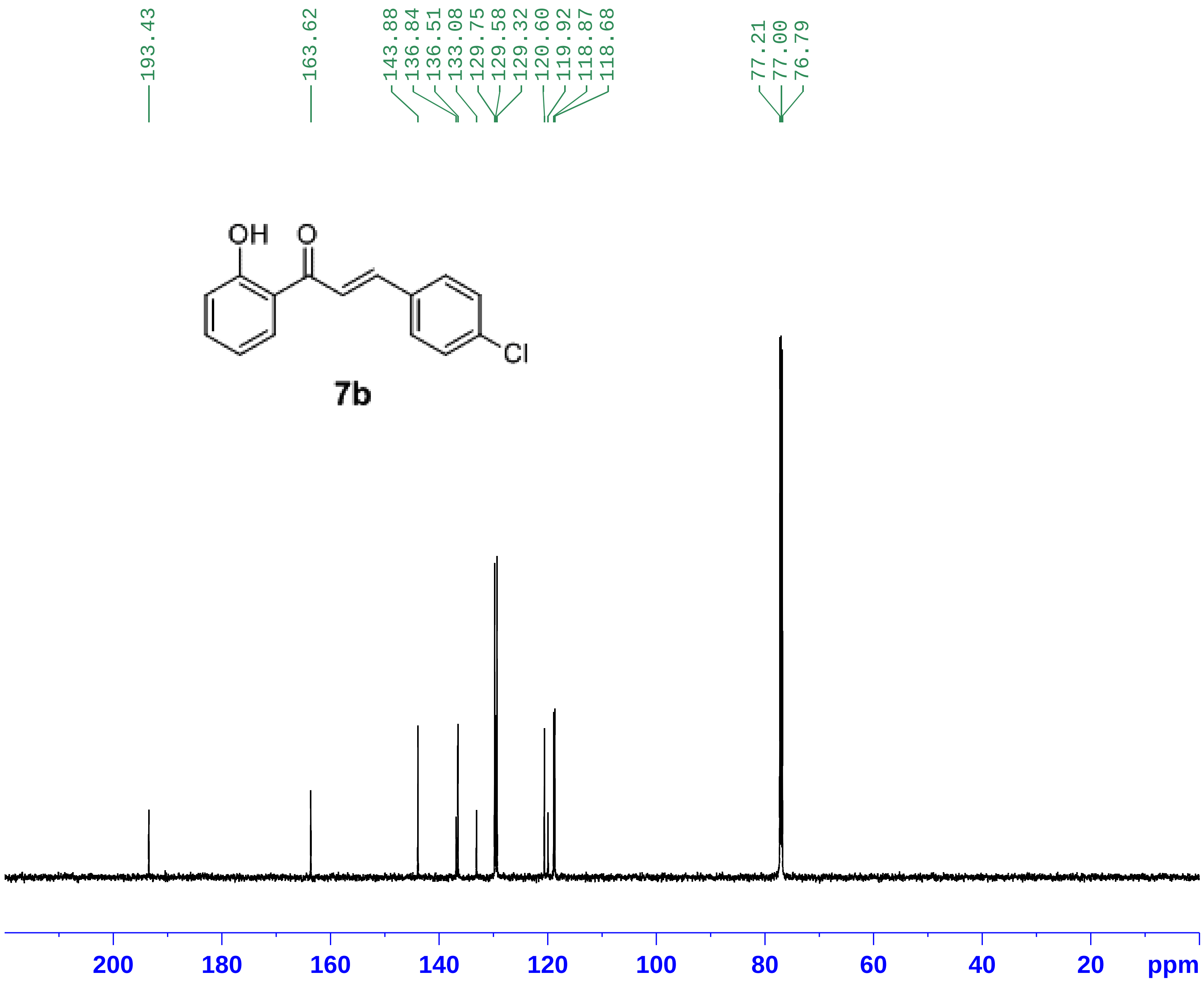


```
Current Data Parameters
NAME              STH1-83
EXPNO                   2
PROCNO                  1

F2 - Acquisition Parameters
Date_            20170606
Time                 9.14
INSTRUM             spect
PROBHD   5 mm PABBO BB-
PULPROG           zgpg30
TD                 65536
SOLVENT            CDCl3
NS                   168
DS                     0
SWH           36231.883 Hz
FIDRES         0.552855 Hz
AQ            0.9043968 sec
RG                 16400
DW                13.800 usec
DE                  6.00 usec
TE                 301.3 K
D1            2.40000010 sec
D11           0.03000000 sec
TD0                    1

======== CHANNEL f1 ========
NUC1                 13C
P1                 10.50 usec
PL1                 2.90 dB
PL1W         48.30935669 W
SFO1        150.9194083 MHz

======== CHANNEL f2 ========
CPDPRG[2          waltz16
NUC2                   1H
PCPD2              90.00 usec
PL2                -0.60 dB
PL12               13.00 dB
PL13               16.00 dB
PL2W         23.07225227 W
PL12W         1.00714028 W
PL13W         0.50476587 W
SFO2        600.1339008 MHz

F2 - Processing parameters
SI                 32768
SF          150.9028127 MHz
WDW                   EM
SSB      0
LB                  3.00 Hz
GB       0
PC                  1.00
```

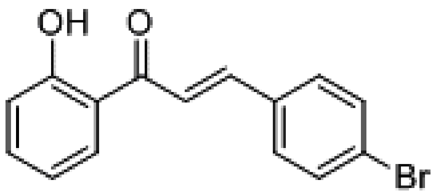

7c

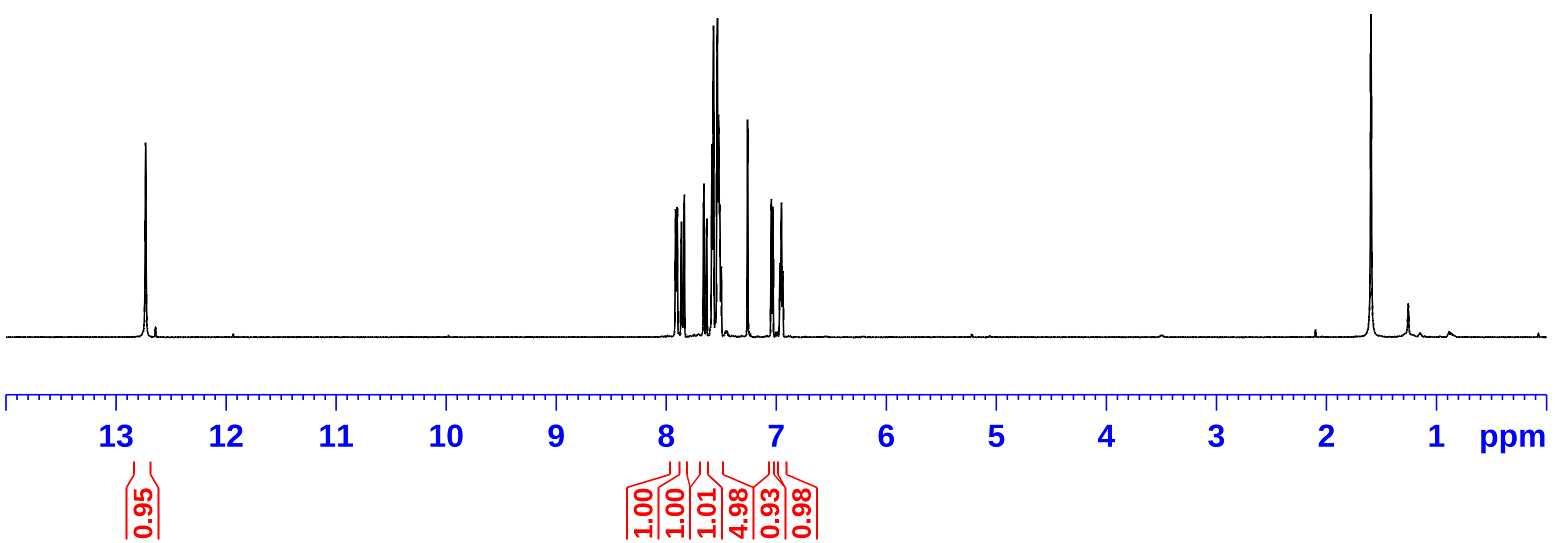


```
Current Data Parameters
NAME              LCW2-95
EXPNO                   1
PROCNO                  1

F2 - Acquisition Parameters
Date_            20150428
Time                16.13
INSTRUM             spect
PROBHD    5 mm TXI 1H/D-
PULPROG              zg30
TD                  32768
SOLVENT             CDCl3
NS                     12
DS                      0
SWH              8992.806 Hz
FIDRES           0.274439 Hz
AQ              1.8219008 sec
RG                    406
DW                 55.600 usec
DE                   6.00 usec
TE                  300.0 K
D1             2.00000000 sec
TD0                     1

======== CHANNEL f1 ========
NUC1                   1H
P1                   9.50 usec
PL1                 -3.30 dB
PL1W          42.96254349 W
SFO1          600.1342009 MHz

F2 - Processing parameters
SI                  16384
SF            600.1300101 MHz
WDW                    no
SSB       0
LB        0 Hz
GB        0
PC                   1.00
```

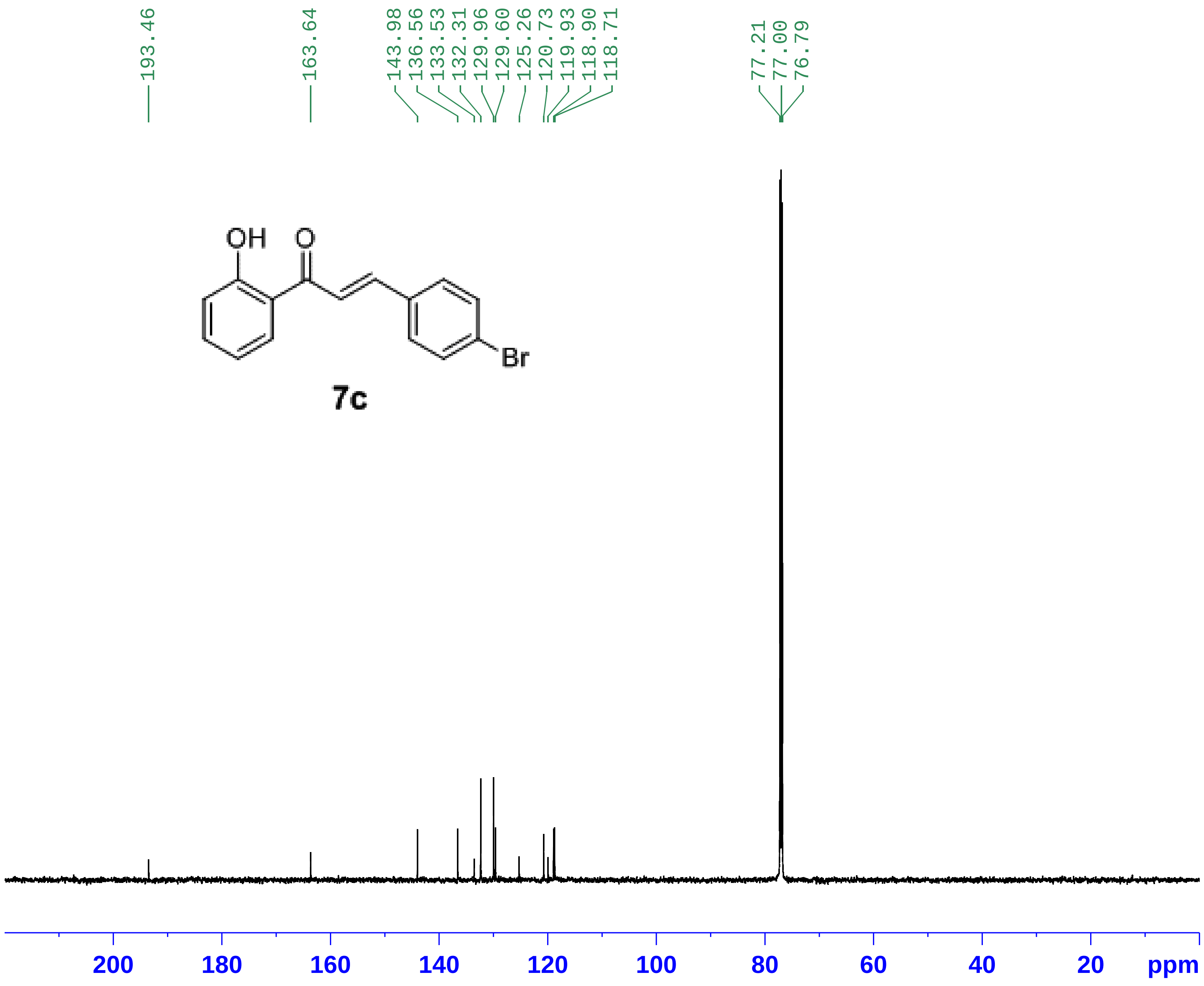

7c

```
Current Data Parameters
NAME              LCW2-95
EXPNO                   2
PROCNO                  1

F2 - Acquisition Parameters
Date_            20150424
Time                11.02
INSTRUM             spect
PROBHD     5 mm PABBO BB-
PULPROG           zgpg30
TD                 65536
SOLVENT            CDCl3
NS                   250
DS                     0
SWH            33333.332 Hz
FIDRES          0.508626 Hz
AQ             0.9830400 sec
RG                 16400
DW                15.000 usec
DE                  6.00 usec
TE                 300.0 K
D1            2.40000010 sec
D11           0.03000000 sec
TD0                    1

======== CHANNEL f1 ========
NUC1                 13C
P1                  9.60 usec
PL1                 2.40 dB
PL1W         54.20399094 W
SFO1         150.9194083 MHz

======== CHANNEL f2 ========
CPDPRG[2         waltz16
NUC2                  1H
PCPD2              90.00 usec
PL2                -4.00 dB
PL12                8.00 dB
PL13               11.00 dB
PL2W         50.47658920 W
PL12W         3.18485737 W
PL13W         1.59620988 W
SFO2         600.1339008 MHz

F2 - Processing parameters
SI                 32768
SF           150.9028100 MHz
WDW                   EM
SSB        0
LB                  3.00 Hz
GB         0
PC                  1.00
```

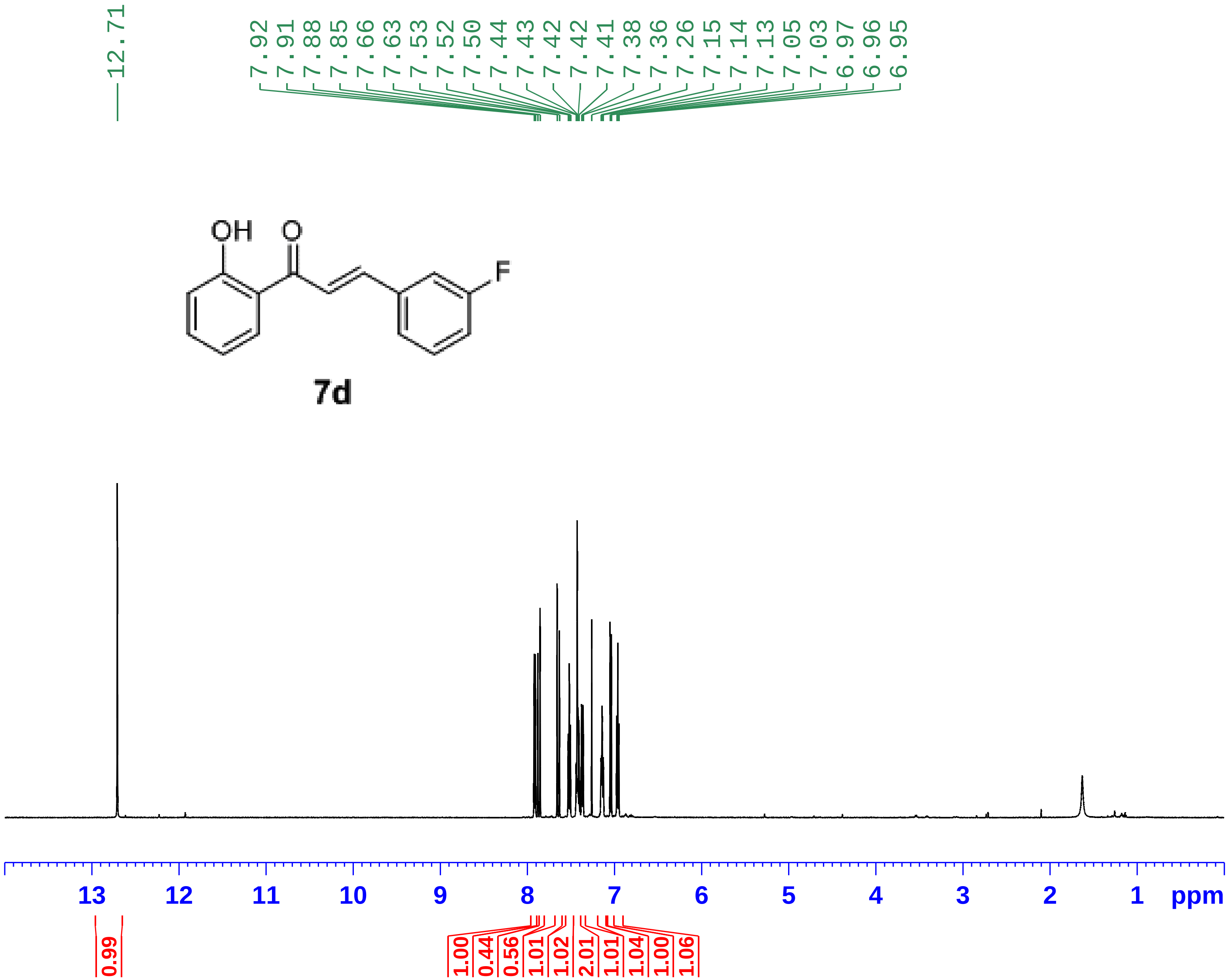

7d

Current Data Parameters
NAME LCW2-87
EXPNO 1
PROCNO 1

F2 - Acquisition Parameters
Date_ 20150417
Time 9.53
INSTRUM spect
PROBHD 5 mm TXI 1H/D-
PULPROG zg30
TD 32768
SOLVENT CDCl3
NS 16
DS 0
SWH 8992.806 Hz
FIDRES 0.274439 Hz
AQ 1.8219008 sec
RG 256
DW 55.600 usec
DE 6.00 usec
TE 300.0 K
D1 2.00000000 sec
TD0 1

======== CHANNEL f1 ========
NUC1 1H
P1 9.50 usec
PL1 -3.30 dB
PL1W 42.96254349 W
SFO1 600.1342009 MHz

F2 - Processing parameters
SI 16384
SF 600.1300105 MHz
WDW EM
SSB 0
LB 0 Hz
GB 0
PC 1.00

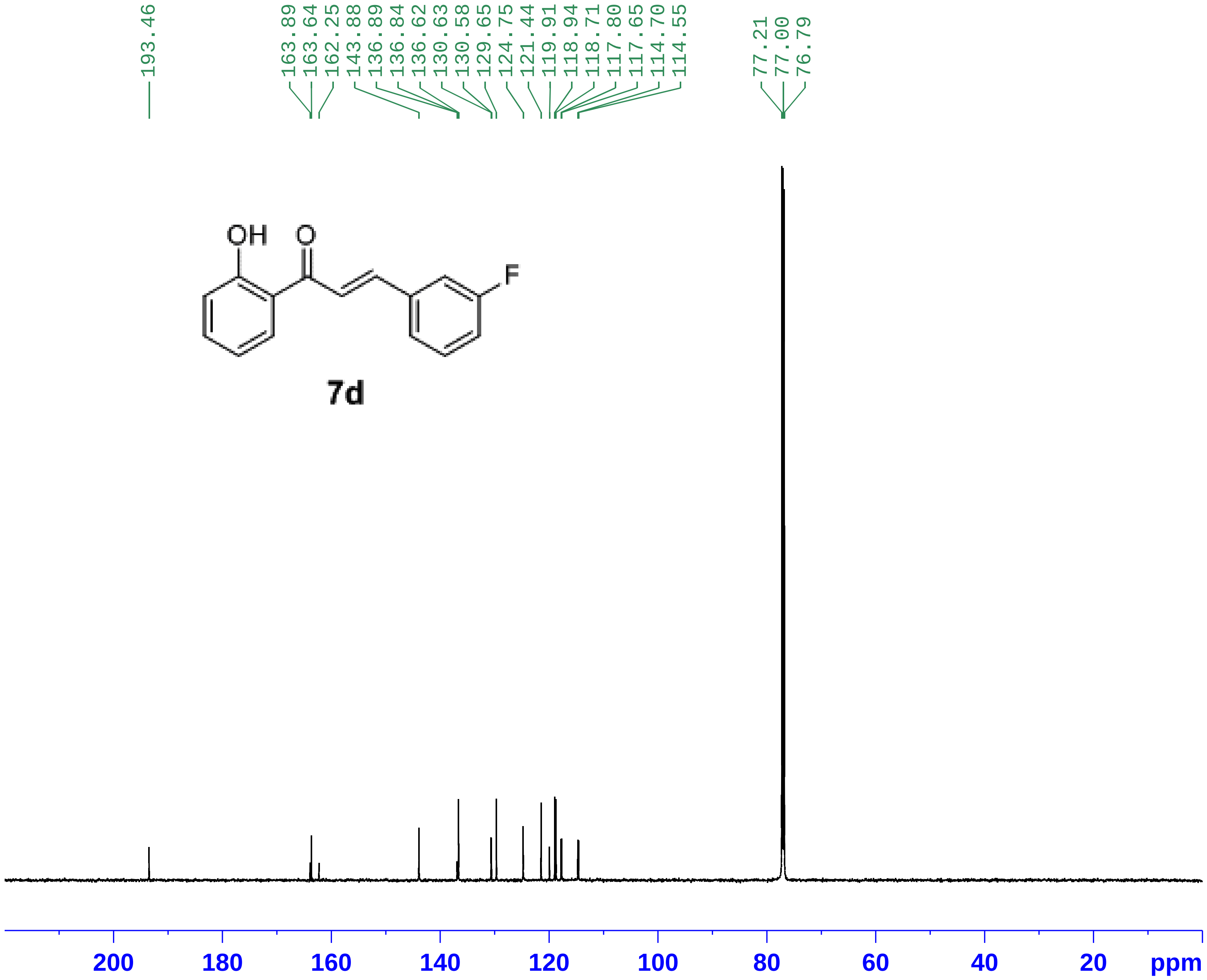

7d

```
Current Data Parameters
NAME              LCW2-87
EXPNO                   2
PROCNO                  1

F2 - Acquisition Parameters
Date_            20150415
Time                15.22
INSTRUM             spect
PROBHD     5 mm PABBO BB-
PULPROG            zgpg30
TD                  65536
SOLVENT             CDCl3
NS                    795
DS                      0
SWH             34013.605 Hz
FIDRES           0.519006 Hz
AQ              0.9633792 sec
RG                  16400
DW                 14.700 usec
DE                   6.00 usec
TE                  300.0 K
D1             2.40000010 sec
D11            0.03000000 sec
TD0                     1

======== CHANNEL f1 ========
NUC1                  13C
P1                   9.60 usec
PL1                  2.40 dB
PL1W          54.20399094 W
SFO1          150.9194083 MHz

======== CHANNEL f2 ========
CPDPRG[2          waltz16
NUC2                   1H
PCPD2               90.00 usec
PL2                 -4.00 dB
PL12                 8.00 dB
PL13                11.00 dB
PL2W          50.47658920 W
PL12W          3.18485737 W
PL13W          1.59620988 W
SFO2          600.1339008 MHz

F2 - Processing parameters
SI                  32768
SF            150.9028099 MHz
WDW                    EM
SSB        0
LB                   3.00 Hz
GB         0
PC                   1.00
```

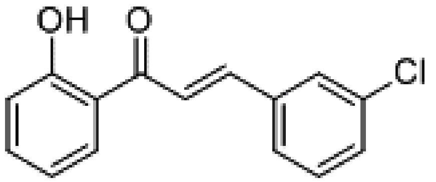

7e

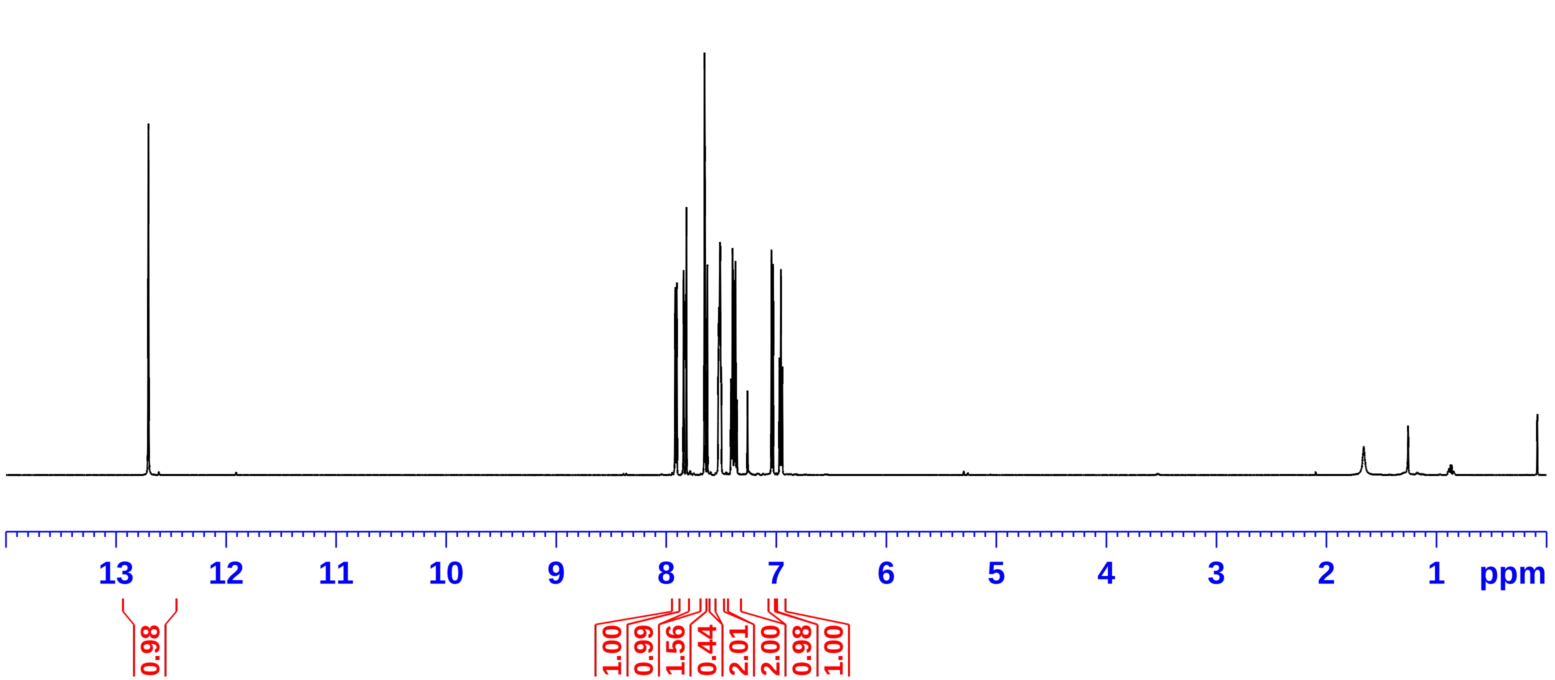

Current Data Parameters
NAME LCW2-89
EXPNO 1
PROCNO 1

F2 - Acquisition Parameters
Date_ 20150420
Time 14.41
INSTRUM spect
PROBHD 5 mm TXI 1H/D-
PULPROG zg30
TD 32768
SOLVENT CDCl3
NS 16
DS 0
SWH 8992.806 Hz
FIDRES 0.274439 Hz
AQ 1.8219008 sec
RG 101
DW 55.600 usec
DE 6.00 usec
TE 300.0 K
D1 2.00000000 sec
TD0 1

======== CHANNEL f1 ========
NUC1 1H
P1 9.50 usec
PL1 -3.30 dB
PL1W 42.96254349 W
SFO1 600.1342009 MHz

F2 - Processing parameters
SI 16384
SF 600.1300104 MHz
WDW no
SSB 0
LB 0 Hz
GB 0
PC 1.00

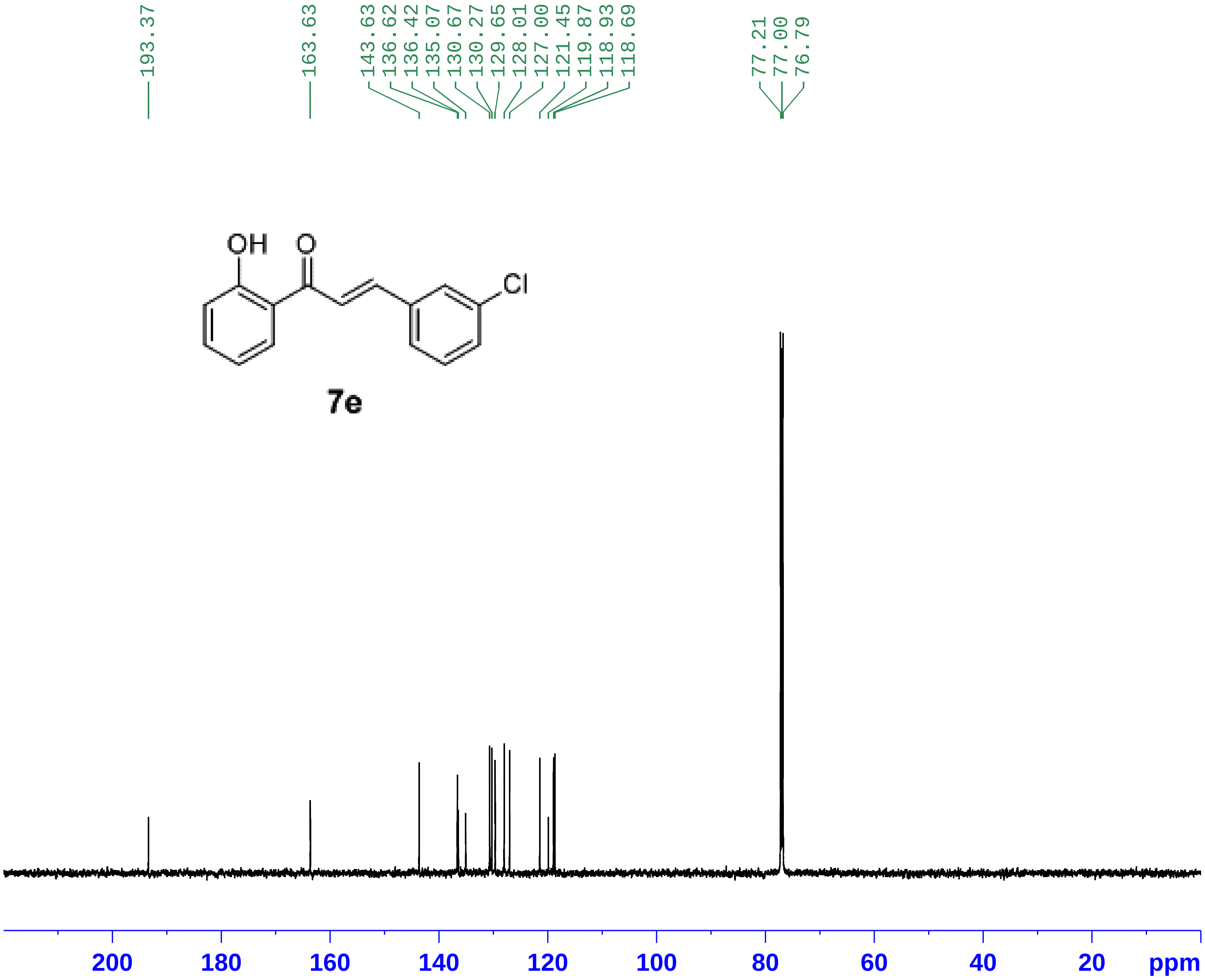

Current Data Parameters
NAME LCW2-89
EXPNO 2
PROCNO 1

F2 - Acquisition Parameters
Date_ 20150416
Time 13.29
INSTRUM spect
PROBHD 5 mm PABBO BB-
PULPROG zgpg30
TD 65536
SOLVENT CDCl3
NS 85
DS 0
SWH 33333.332 Hz
FIDRES 0.508626 Hz
AQ 0.9830400 sec
RG 16400
DW 15.000 usec
DE 6.00 usec
TE 300.0 K
D1 2.40000010 sec
D11 0.03000000 sec
TD0 1

======== CHANNEL f1 ========
NUC1 13C
P1 9.40 usec
PL1 2.40 dB
PL1W 54.20399094 W
SFO1 150.9194083 MHz

======== CHANNEL f2 ========
CPDPRG[2 waltz16
NUC2 1H
PCPD2 90.00 usec
PL2 -4.00 dB
PL12 11.30 dB
PL13 14.30 dB
PL2W 50.47658920 W
PL12W 1.48966968 W
PL13W 0.74660343 W
SFO2 600.1339008 MHz

F2 - Processing parameters
SI 32768
SF 150.9028125 MHz
WDW EM
SSB 0
LB 3.00 Hz
GB 0
PC 1.00

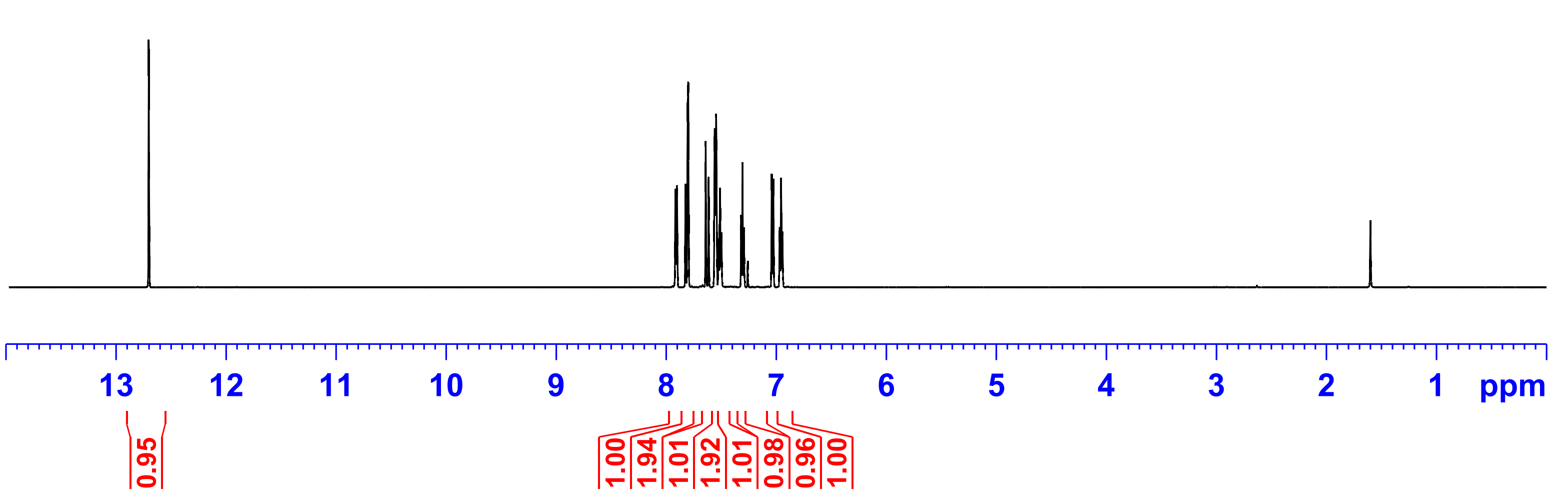

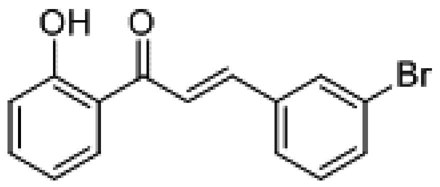

7f

BRUKER

```
Current Data Parameters
NAME              STH1-115
EXPNO                    1
PROCNO                   1

F2 - Acquisition Parameters
Date_             20170901
Time                 10.48
INSTRUM              spect
PROBHD    5 mm TXI 1H/D-
PULPROG               zg30
TD                   32768
SOLVENT              CDCl3
NS                      16
DS                       0
SWH            8389.262 Hz
FIDRES         0.256020 Hz
AQ            1.9529728 sec
RG                     203
DW               59.600 usec
DE                 6.00 usec
TE                300.0 K
D1           2.00000000 sec
TD0                      1

======== CHANNEL f1 ========
NUC1                    1H
P1                 9.30 usec
PL1               -1.50 dB
PL1W        28.38507080 W
SFO1       600.1342009 MHz

F2 - Processing parameters
SI                   16384
SF         600.1300108 MHz
WDW                     no
SSB      0
LB       0 Hz
GB       0
PC                    1.00
```

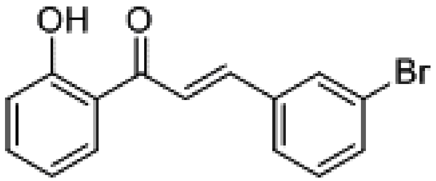

7f

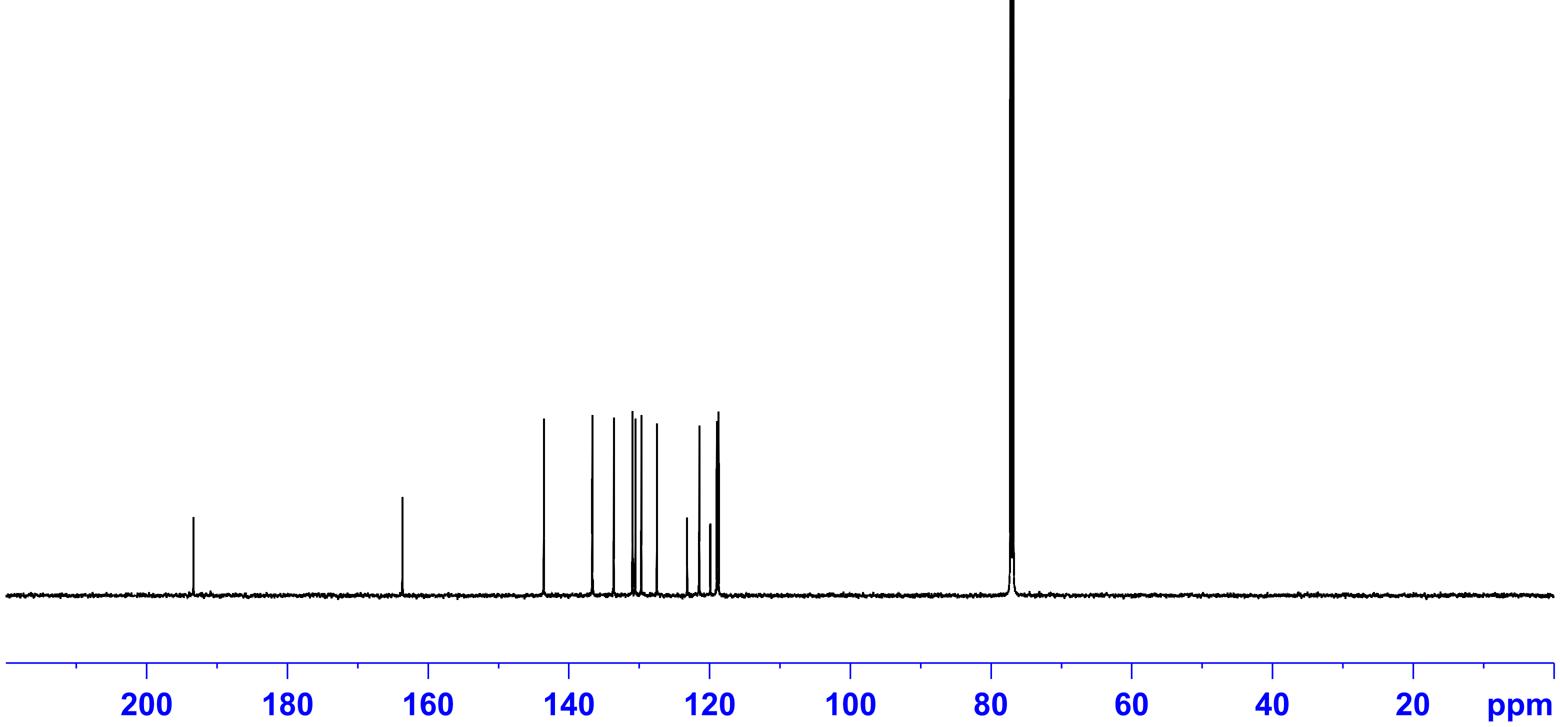


```
Current Data Parameters
NAME              STH1-115
EXPNO                    2
PROCNO                   1

F2 - Acquisition Parameters
Date_             20170901
Time                 13.42
INSTRUM              spect
PROBHD     5 mm PABBO BB-
PULPROG            zgpg30
TD                   65536
SOLVENT              CDCl3
NS                     483
DS                       0
SWH          36231.883 Hz
FIDRES        0.552855 Hz
AQ           0.9043968 sec
RG                   16400
DW              13.800 usec
DE               6.00 usec
TE                298.0 K
D1          2.40000010 sec
D11         0.03000000 sec
TD0                      1

======== CHANNEL f1 ========
NUC1                   13C
P1              10.50 usec
PL1                2.90 dB
PL1W       48.30935669 W
SFO1       150.9194083 MHz

======== CHANNEL f2 ========
CPDPRG[2           waltz16
NUC2                    1H
PCPD2           90.00 usec
PL2               -0.60 dB
PL12              13.00 dB
PL13              16.00 dB
PL2W       23.07225227 W
PL12W       1.00714028 W
PL13W       0.50476587 W
SFO2       600.1339008 MHz

F2 - Processing parameters
SI                   32768
SF         150.9028148 MHz
WDW                     EM
SSB        0
LB                 3.00 Hz
GB         0
PC                    1.00
```

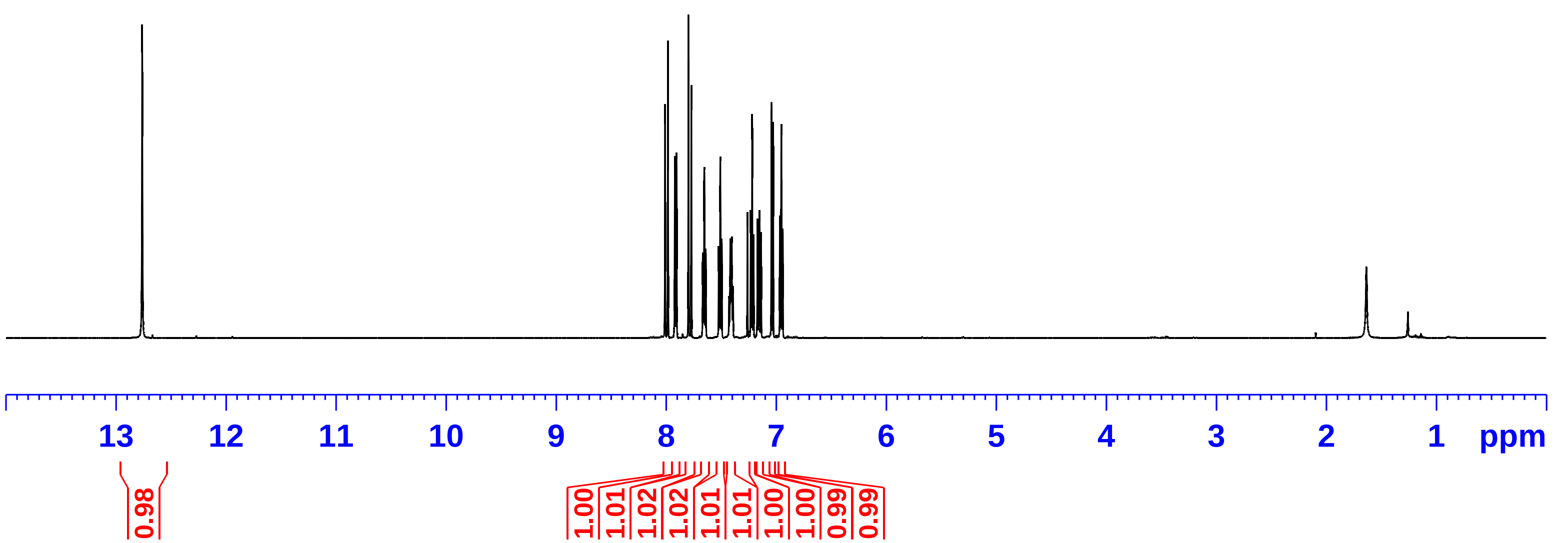

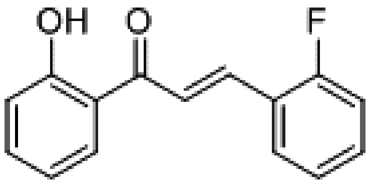

7g

BRUKER

Current Data Parameters
NAME LCW2-102
EXPNO 1
PROCNO 1

F2 - Acquisition Parameters
Date_ 20150513
Time 11.15
INSTRUM spect
PROBHD 5 mm TXI 1H/D-
PULPROG zg30
TD 32768
SOLVENT CDCl3
NS 16
DS 0
SWH 8992.806 Hz
FIDRES 0.274439 Hz
AQ 1.8219008 sec
RG 114
DW 55.600 usec
DE 6.00 usec
TE 300.0 K
D1 2.00000000 sec
TD0 1

======== CHANNEL f1 ========
NUC1 1H
P1 9.50 usec
PL1 -3.30 dB
PL1W 42.96254349 W
SFO1 600.1342009 MHz

F2 - Processing parameters
SI 16384
SF 600.1300105 MHz
WDW EM
SSB 0
LB 0 Hz
GB 0
PC 1.00

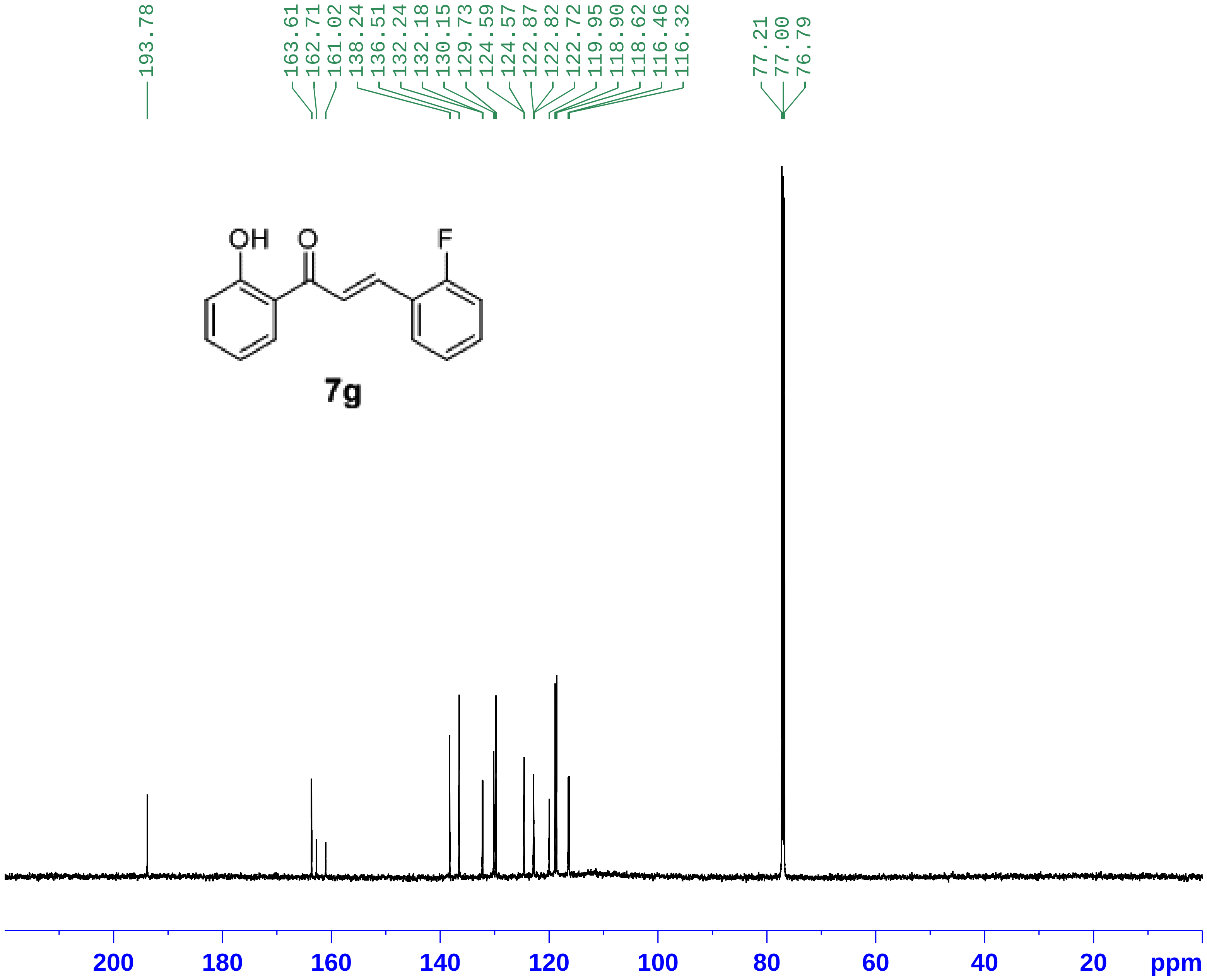

7g

```
Current Data Parameters
NAME              LCW2-102
EXPNO                    2
PROCNO                   1

F2 - Acquisition Parameters
Date_             20150513
Time                 11.50
INSTRUM              spect
PROBHD    5 mm TXI 1H/D-
PULPROG            zgpg30
TD                   65536
SOLVENT              CDCl3
NS                     553
DS                       0
SWH            36231.883 Hz
FIDRES          0.552855 Hz
AQ             0.9043968 sec
RG                   14600
DW                  13.800 usec
DE                    6.00 usec
TE                   300.0 K
D1              2.40000010 sec
D11             0.03000000 sec
TD0                      1

======== CHANNEL f1 ========
NUC1                   13C
P1                   10.50 usec
PL1                  -4.00 dB
PL1W          236.60900879 W
SFO1          150.9194083 MHz

======== CHANNEL f2 ========
CPDPRG[2           waltz16
NUC2                    1H
PCPD2                90.00 usec
PL2                  -3.30 dB
PL12                 13.00 dB
PL13                 16.00 dB
PL2W           42.96254349 W
PL12W           1.00714028 W
PL13W           0.50476587 W
SFO2          600.1339008 MHz

F2 - Processing parameters
SI                   32768
SF            150.9028128 MHz
WDW                     EM
SSB       0
LB                    3.00 Hz
GB        0
PC                    1.00
```

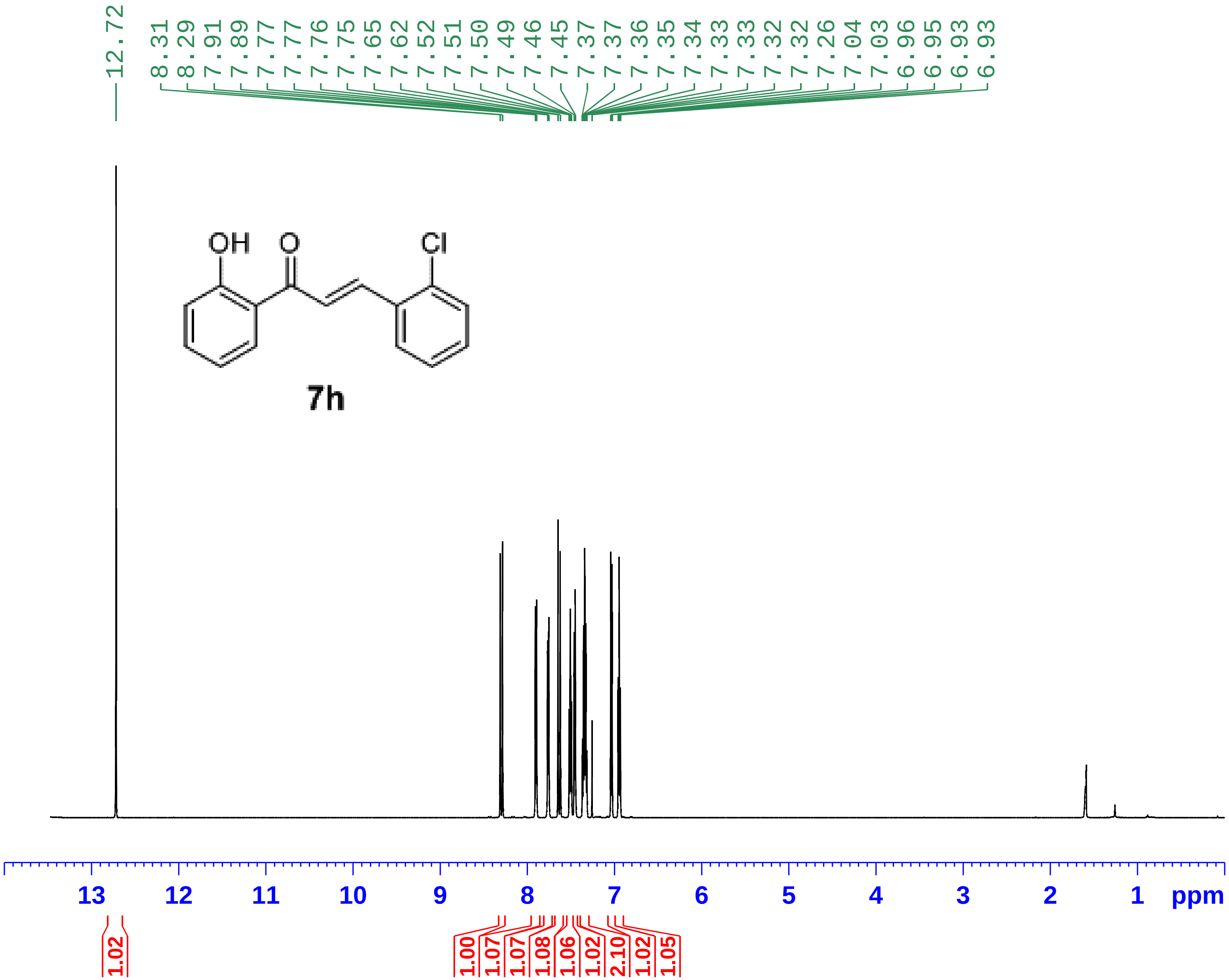


```
Current Data Parameters
NAME              STH1-82
EXPNO                   1
PROCNO                  1

F2 - Acquisition Parameters
Date_            20170606
Time                10.40
INSTRUM             spect
PROBHD    5 mm TXI 1H/D-
PULPROG              zg30
TD                  32768
SOLVENT             CDCl3
NS                     16
DS                      0
SWH              8992.806 Hz
FIDRES           0.274439 Hz
AQ              1.8219008 sec
RG                    181
DW                 55.600 usec
DE                   6.00 usec
TE                  300.0 K
D1             2.00000000 sec
TD0                     1

======== CHANNEL f1 ========
NUC1                   1H
P1                   8.50 usec
PL1                 -1.80 dB
PL1W          30.41515160 W
SFO1         600.1336008 MHz

F2 - Processing parameters
SI                  16384
SF           600.1300113 MHz
WDW                    no
SSB       0
LB        0 Hz
GB        0
PC                   1.00
```

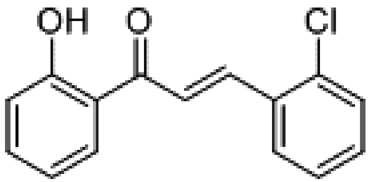

7h

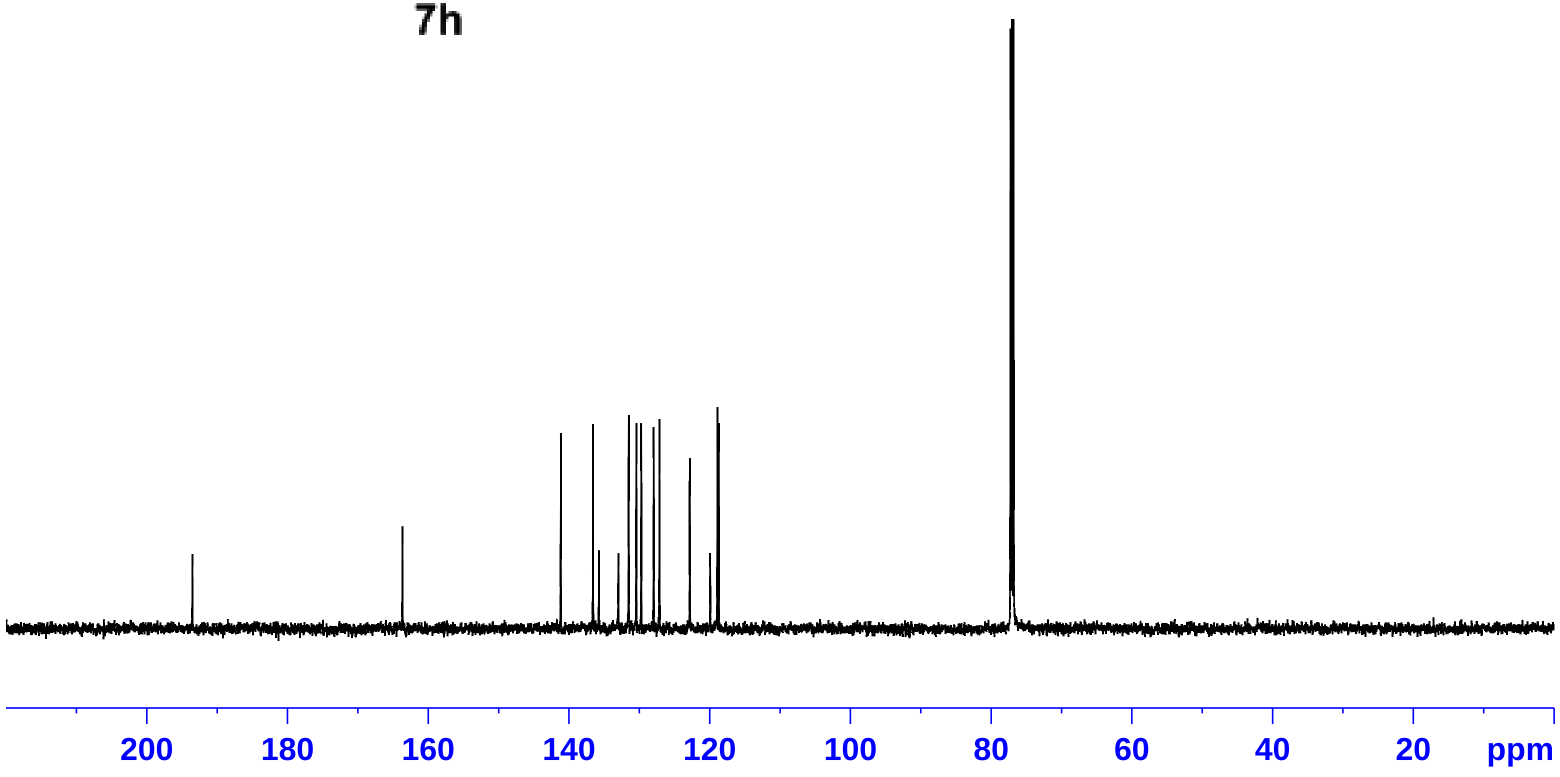

Current Data Parameters
NAME STH1-82
EXPNO 2
PROCNO 1

F2 - Acquisition Parameters
Date_ 20170606
Time 8.59
INSTRUM spect
PROBHD 5 mm PABBO BB-
PULPROG zgpg30
TD 65536
SOLVENT DMSO
NS 115
DS 0
SWH 36231.883 Hz
FIDRES 0.552855 Hz
AQ 0.9043968 sec
RG 16400
DW 13.800 usec
DE 6.00 usec
TE 301.1 K
D1 2.40000010 sec
D11 0.03000000 sec
TD0 1

======== CHANNEL f1 ========
NUC1 13C
P1 10.50 usec
PL1 2.90 dB
PL1W 48.30935669 W
SFO1 150.9194083 MHz

======== CHANNEL f2 ========
CPDPRG[2 waltz16
NUC2 1H
PCPD2 90.00 usec
PL2 -0.60 dB
PL12 13.00 dB
PL13 16.00 dB
PL2W 23.07225227 W
PL12W 1.00714028 W
PL13W 0.50476587 W
SFO2 600.1339008 MHz

F2 - Processing parameters
SI 32768
SF 150.9027317 MHz
WDW EM
SSB 0
LB 3.00 Hz
GB 0
PC 1.00

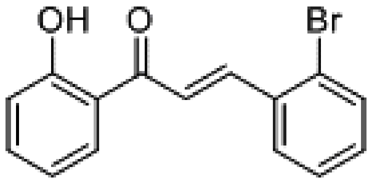

7i

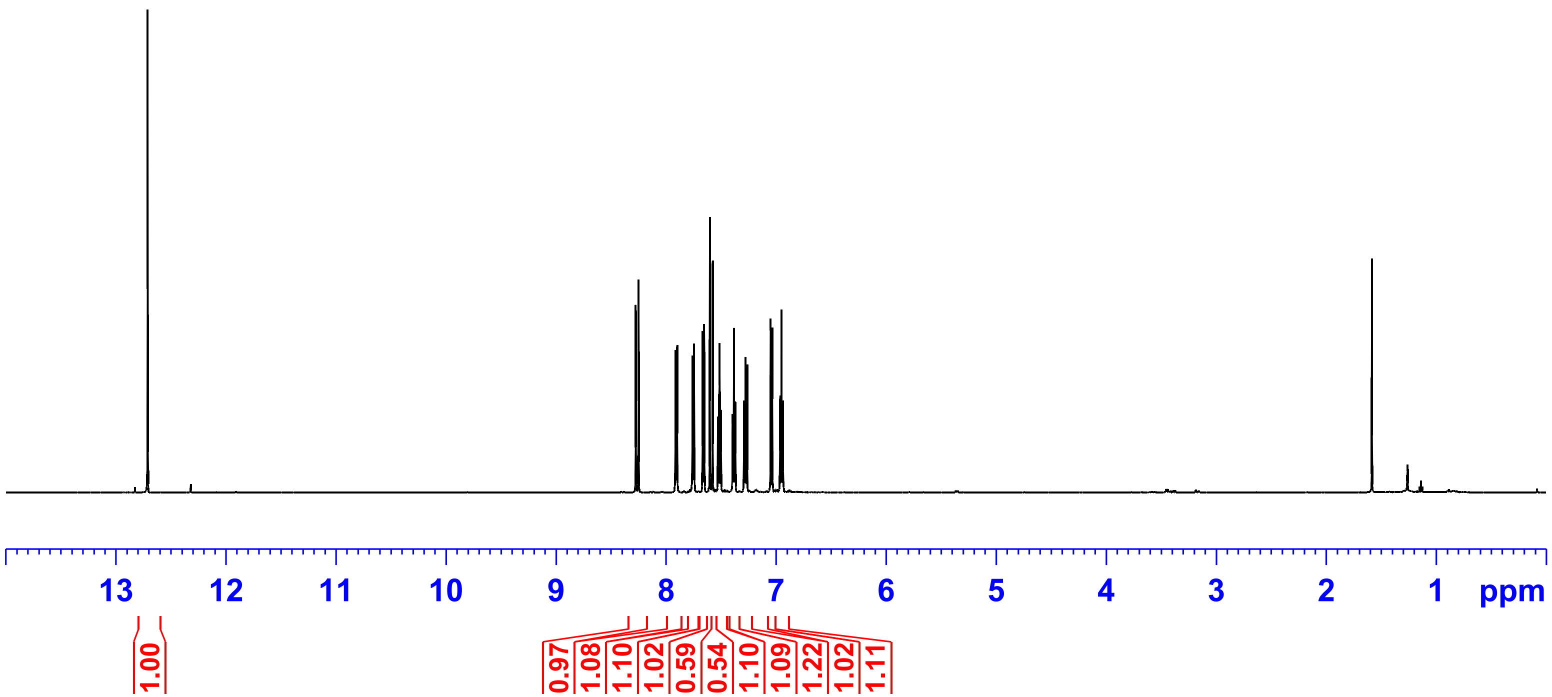

Current Data Parameters
NAME STH1-81
EXPNO 1
PROCNO 1

F2 - Acquisition Parameters
Date_ 20170526
Time 14.06
INSTRUM spect
PROBHD 5 mm TXI 1H/D-
PULPROG zg30
TD 32768
SOLVENT CDCl3
NS 16
DS 0
SWH 9615.385 Hz
FIDRES 0.293438 Hz
AQ 1.7039360 sec
RG 203
DW 52.000 usec
DE 6.00 usec
TE 300.0 K
D1 2.00000000 sec
TD0 1

======== CHANNEL f1 ========
NUC1 1H
P1 9.50 usec
PL1 -3.30 dB
PL1W 42.96254349 W
SFO1 600.1342009 MHz

F2 - Processing parameters
SI 16384
SF 600.1300098 MHz
WDW no
SSB 0
LB 0 Hz
GB 0
PC 1.00

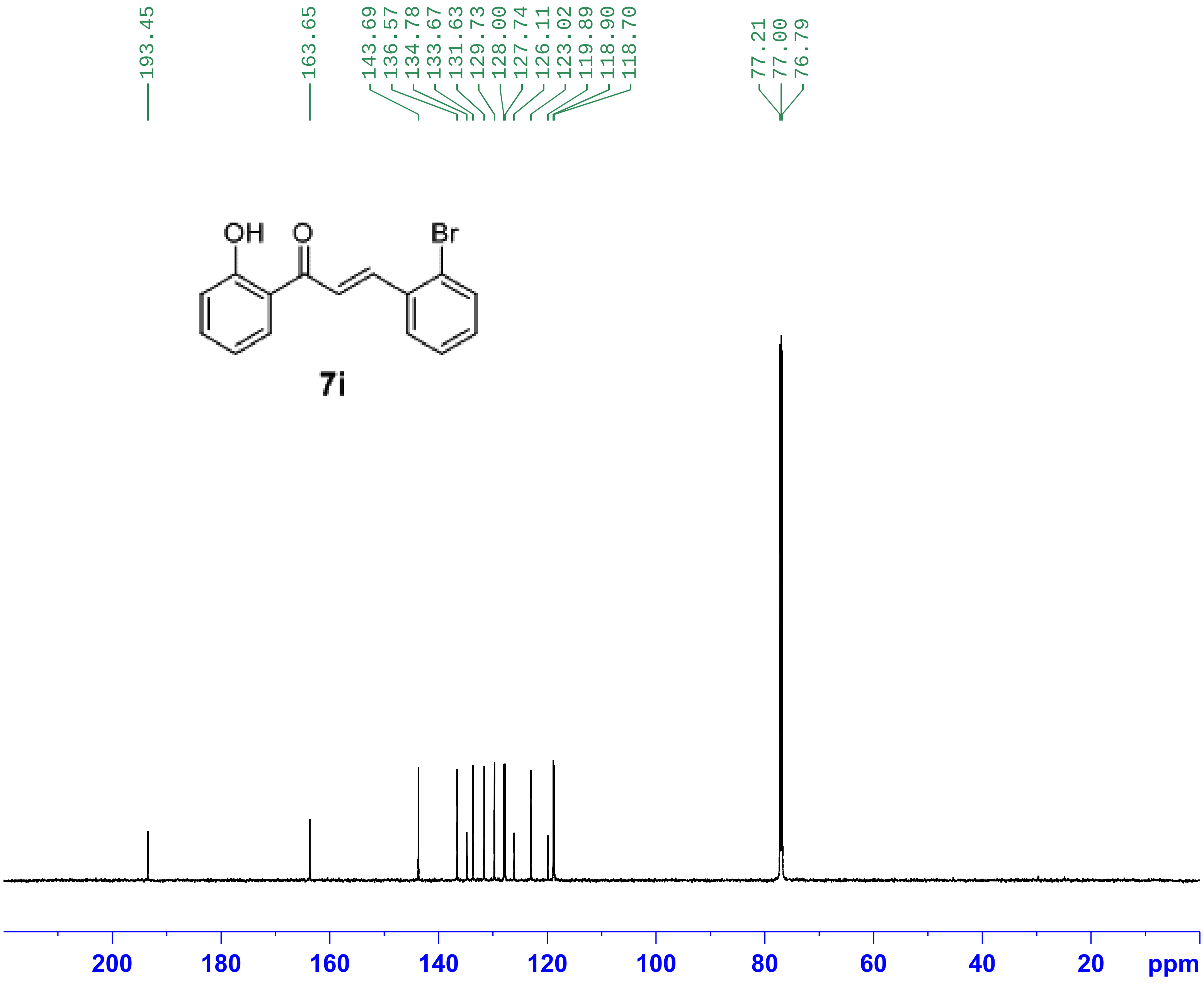

Current Data Parameters
NAME STH1-81
EXPNO 2
PROCNO 1

F2 - Acquisition Parameters
Date_ 20170526
Time 9.29
INSTRUM spect
PROBHD 5 mm PABBO BB-
PULPROG zgpg30
TD 65536
SOLVENT CDCl3
NS 767
DS 0
SWH 33333.332 Hz
FIDRES 0.508626 Hz
AQ 0.9830400 sec
RG 16400
DW 15.000 usec
DE 6.00 usec
TE 307.2 K
D1 2.40000010 sec
D11 0.03000000 sec
TD0 1

======== CHANNEL f1 ========
NUC1 13C
P1 10.50 usec
PL1 2.90 dB
PL1W 48.30935669 W
SFO1 150.9194083 MHz

======== CHANNEL f2 ========
CPDPRG[2 waltz16
NUC2 1H
PCPD2 90.00 usec
PL2 -0.60 dB
PL12 13.00 dB
PL13 16.00 dB
PL2W 23.07225227 W
PL12W 1.00714028 W
PL13W 0.50476587 W
SFO2 600.1339008 MHz

F2 - Processing parameters
SI 32768
SF 150.9028120 MHz
WDW EM
SSB 0
LB 3.00 Hz
GB 0
PC 1.00
